# Base-Catalyzed Pathway Towards Isocyanate Derivatives of Silsesquioxanes

**DOI:** 10.3390/ijms26167769

**Published:** 2025-08-12

**Authors:** Kamil Hanek, Monika Wałęsa-Chorab, Patrycja Żak

**Affiliations:** Faculty of Chemistry, Adam Mickiewicz University in Poznań, Uniwersytetu Poznańskiego St. 8, 61-614 Poznań, Poland; kamil.hanek@amu.edu.pl (K.H.); monika.walesa-chorab@amu.edu.pl (M.W.-C.)

**Keywords:** silsesquioxanes, isocyanates, nanomaterials, thermal stability, photophysical properties

## Abstract

Easily accessible and inexpensive potassium carbonate (K_2_CO_3_) has been applied as the base-catalyst for the synthesis of novel classes of functionalized nanomaterials. This eco-friendly approach has been proven to be effective for a wide range of substrates, leading to nine isocyanate derivatives of silsesquioxanes (SQs) with yields exceeding 90% in mild and transition metal-free conditions. The application potential of chosen products was assessed on the basis of thermogravimetric analyses and photochemical measurements.

## 1. Introduction

**Figure 1:**
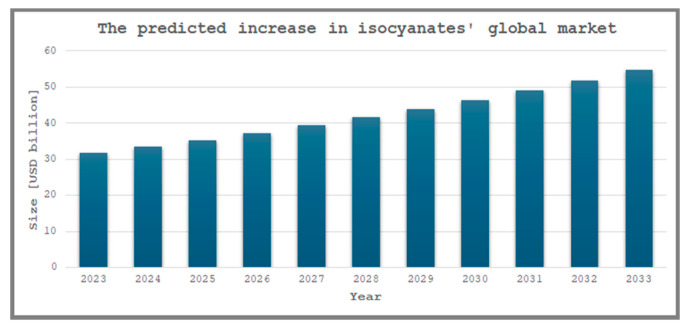
Expected increase in global market of isocyanates.

Isocyanates are a group of reactive chemical compounds widely used in the industry. They can be applied in the production of spray paints [1], polyurethanes [2], biosensors [3], green fertilizers [4], and isolation foams [5]. Isocyanate and their derivatives can also find some applications in the pharmaceutical and medicinal industries, as they are present in many drugs [6,7,8], e.g., in those against bladder cancer [9], in those with cardio-protective effects [10], in fluorescent sensors [11], in bioimaging agents [12,13], in enzyme inhibitors [14], and in photochemical release systems [15]. In the field of chemical technology, these compounds have found applications as photocatalysts [16], heavy metal photosensors [17], and photochemical cryogenic matrices [18]. Moreover, they play a particularly important role in novel printing processes, e.g., photolithography, micropatterning, and 3D printing photo-initiators [19,20,21]. Because of their wide applications in areas of crucial importance, it is predicted that, in the near future, their global market will note a significant increase (Figure 1).

Despite many advantages, isocyanates struggle with some drawbacks that could hinder development in this area of chemistry. The most important issue is their relatively low thermal stability and photostability [22,23]. Polyurethanes made from typical isocyanates as well as polymers based on S-thiazole rings have a tendency to decompose at relatively mild temperatures and under the influence of light [24,25]. Another issue is their affinity to water. As they are used as spray paints, isolation foams, or humidity sensors, much effort is directed towards their modification to obtain materials that would offer greater thermal stability and water resistance.

A possible solution to this problem could be the incorporation of polyhedral oligomeric silsesquioxanes (SQs) into the isocyanates’ structures. SQs represent a broad class of materials of well-defined, nanometric cage entities of the general formula [RSiO_1.5_]_n_. They are completely non-toxic, robust, and thermally stable moieties based on the siloxane framework [26]. Because of their nature, they can be easily adjusted to serve many different applications [27,28,29,30,31]. In contemporary science and industry, SQs are mostly used as drug nanocarriers to which drugs are bonded through physical or chemical interactions [32,33,34] or as the components of hydrogel and band-aids [35,36,37]. As ascertained from reviewing the literature, SQs have many applications in common with isocyanates and their derivatives. They can also be a component in spray-coats [38], polyurethanes [39], or water adsorbents [40]. That is why the use of SQs in the synthesis of practical materials based on isocyanates can significantly improve the properties of final products.

Here, we present a simple yet effective procedure for the base-catalyzed synthesis of isocyanate derivatives incorporated into the SQs framework that is fully consistent with green chemistry principles. The proposed protocol was realized for three different SQ moieties and proven to be effective for obtaining novel classes of hybrid materials. Because of the low thermal stability and photostability of isocyanates and their derivatives as well as their many photochemical applications, the aim of this study is also to assess the thermal and photochemical properties of final materials and to compare them with those of the initial substrates.

## 2. Results and Discussion

### 2.1. Design and Optimization of the Reaction System

We started our study with the model reaction between equimolar amounts of mercapto-propylisobutyl POSS (**SQ-SH**) and 2-chloroethyl isothiocyanate (**1a**) in the presence of 0.6 equiv. of commercially available potassium carbonate (K_2_CO_3_). The process was carried out at the boiling point of toluene and the ^1^H NMR spectra of the post-reaction mixture revealed the formation of the desired five-membered N,S-heterocyclic thiazoline (**P1**) with a yield of 85%. The reaction also led to the formation of ca. 15% of a by-product, which is formed by the condensation of SQ thiol. This achievement prompted us to perform an additional test in order to determine the optimal conditions for **1a** functionalization. The results are summarized in Table 1, in which the SQ skeleton is depicted as a cube. In order to increase the clarity of the results, such a simplification was applied to all structures presented in the paper.

The results gathered in Table 1 reveal that the outcome of the tested process largely depends on the temperature. Among the temperatures tested, the optimal performance of the reaction was observed at 60 °C (Table 1, Entry 12). When the process was carried out at higher temperatures, the formation of SQ-based disulfide was detected (Table 1, Entries 1–3). This is the result of the partial thermolysis of the thiol, leading to the formation of thiyl radicals, which can react with each other to form a disulfide bond in a dimerization process [41,42]. On the other hand, lowering the temperature below 60 °C led to a significant reduction in the conversion of both reagents and a decrease in the yield of **P1** (Table 1, Entries 5–6 and 9). The test reactions performed in different solvents confirmed that the process can be efficiently carried out in toluene, acetone, isobutyl ketone (MIBK), and isopropanol (Table 1, Entries 4, 10 and 16–17), while the use of ethyl acetate (EtOAc) led to a decrease in the substrates conversion to only 10%. Taking into account the price, ecological aspects, and boiling point, acetone was selected as the most suitable solvent for further research. The catalytic screening also demonstrated that the base loading affected the efficiency of the process, because its lowering below 0.6 equiv. led to a reduction in the yield of **P1** (Table 1, Entries 10–11). The experiments carried out in the absence of a salt did not afford any products, even though the reaction time was extended to 72 h (Table 1, Entry 15). This result has confirmed that a base is indispensable for effective reaction course. Surprisingly, we did not observe significant differences between the processes performed in the presence of air and under the inert atmosphere (Table 1, Entry 4 vs. Entry 7). Finally, the impact of the reaction time was checked (Entries 9 and 11–14). It has been proven that 5 h is the shortest time that allows the full conversion of the reagents. After all of the experiments, the conditions specified in Entry 12 were concluded as optimal.

### 2.2. Scope of the Reaction

**Figure 2:**
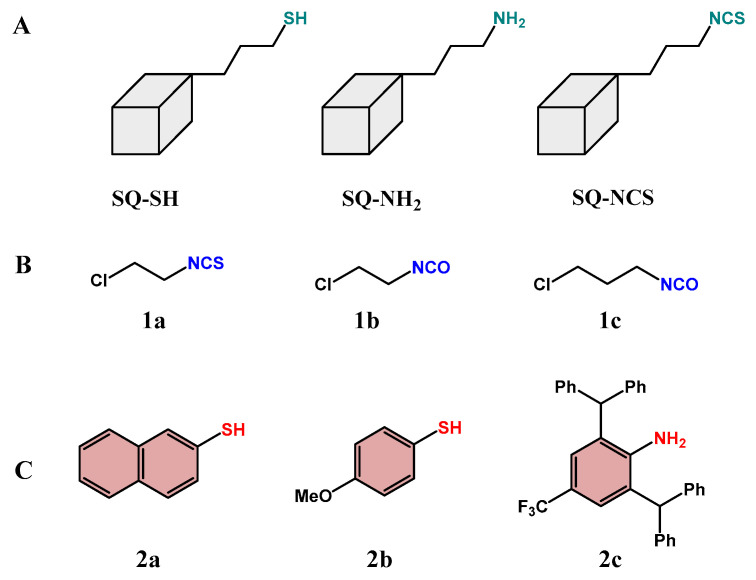
Substrates tested: (**A**) SQs, (**B**) isocyanates, and (**C**) thiol and amine.

Following our successful initial findings, we decided to examine the scope and limitations of this catalytic system. Thus, we probed the reactivity of three different SQs (Figure 2A) to commercially available chloroalkyl isocyanates (Figure 2B) as well as selected nucleophiles (Figure 2C).

We turned our attention to the functionalization of the compounds of this kind because of their great application potential. Our goal was not to synthesize a wide range of compounds, but to indicate the possibilities of improving the properties of the commonly used materials.

**Scheme 1:**
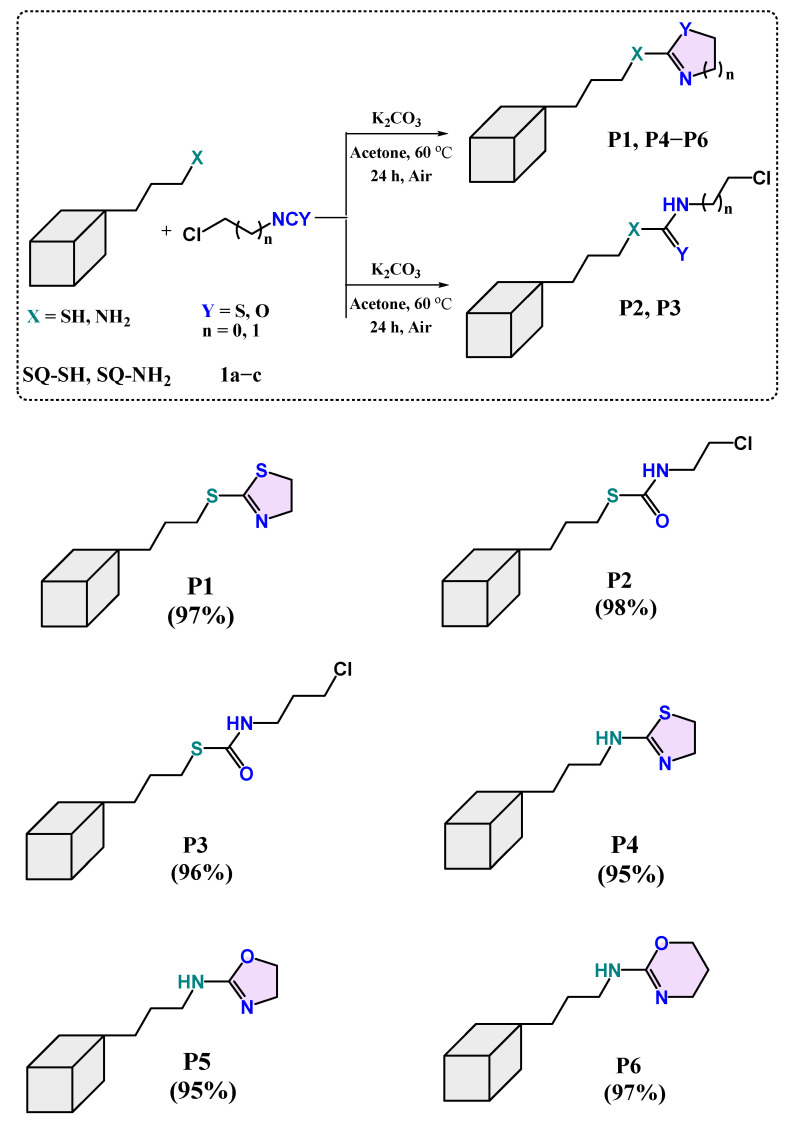
Functionalization of SQs (SQ-SH, SQ-NH_2_) with 1**a**–**c**.

In the first series of experiments presented in Scheme 1, we probed the reactivity of two commercially available SQs (**SQ**-**SH**, **SQ**-**NH_2_**) toward chloroalkyl isothiocyanate (**1a**) and chloroalkyl isocyanates (**1b**,**c**). For all tested substrates, we achieved quantitative yields and complete chemoselectivity towards the formation of the single products. Regardless of the combination of reagents used, we obtained five- or six-membered heterocyclic derivatives (**P1** and **P4**–**P6**). Only in the reactions between **SQ-SH** and both isocyanates (**1b**,**c**) were the final materials identified as S-thiocarbamates (**P2**, **P3**). We did not observe the formation of other products of competitive reactions for any of the reagents studied, which proves that the synthetic protocol works effectively with different moieties.

**Scheme 2:**
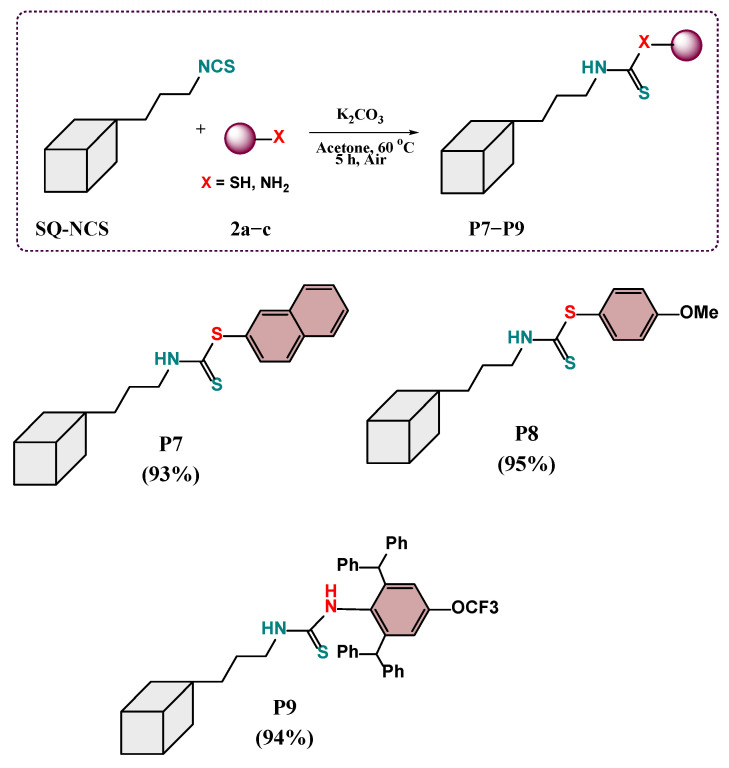
Functionalization of **SQ-NCS** with **2a**–**c**.

In the optimized reaction systems, we have also checked the possibility of using the **SQ-NCS** and selected nucleophiles (**2a**–**c**). As SQs containing the NCS group were commercially unavailable, we started this stage of research with its synthesis carried out according to the methodology described in the literature. The results are presented in Scheme 2. The proposed method can be successfully applied for the tested reagents, because all the products were obtained with excellent isolated yields (>94%) in very mild conditions and with full atom economy. Due to the lack of terminal chlorine atom in iso(thio)cyanate, in all cases, we observed the selective formation of linear products in the form of either dithiocarbamate (**P7**–**P8**) or derivatives of thiourea (**P9**). No meaningful differences in the efficiency and selectivity of the process for the applied reagents was noted.

Within this study, in accordance with the standard practice of our laboratory, all catalytic tests were repeated three times and the obtained results indicated the high reproducibility of the method. All products were isolated and characterized by spectroscopic methods (See ESI for details). These materials are air-stable, which makes them very attractive for further applications. The presented derivatives are new compounds that have never been previously described in the literature.

### 2.3. The Preparative Scale of Synthesis of Product P1

**Scheme 3:**
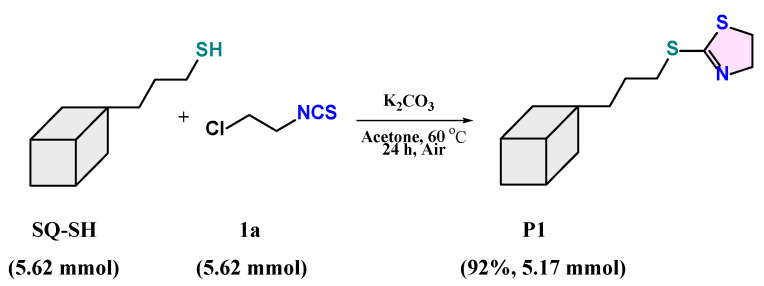
Scaled-up synthesis of **P1**.

To demonstrate the synthetic utility of the designed protocol, a gram scale reaction of **SQ-SH** with **1a** was performed (Scheme 3). The result obtained makes it clear that the proposed methodology has significant application potential.

### 2.4. Proposed Mechanisms

**Scheme 4:**
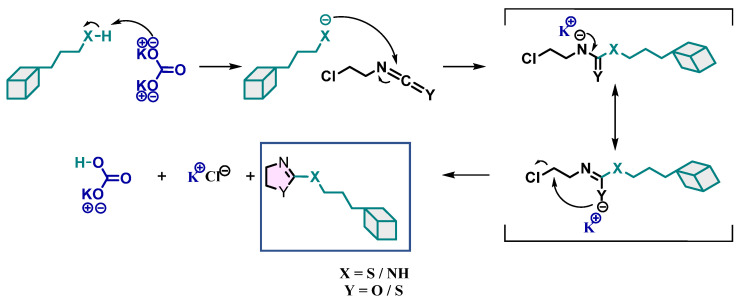
Proposed mechanism of five-membered N,S-heterocyclic 1,3-thiazolines formation.

Based on the literature and our previous work, we propose mechanistic pathways of the reactions leading to the obtained products. In the case of cyclic products [43,44,45], the reaction is based on the addition–substitution pattern. The base in the form of potassium carbonate acts as a proton acceptor. The thiolate anion or amine undergoes the addition to the iso(thio)cyanate moiety, creating intermediate (carbonimidodithioate or thiourea derivative). The intramolecular nucleophilic reaction takes place, eliminating the terminal chlorine atom and giving the cyclic product (Scheme 4).

**Scheme 5:**
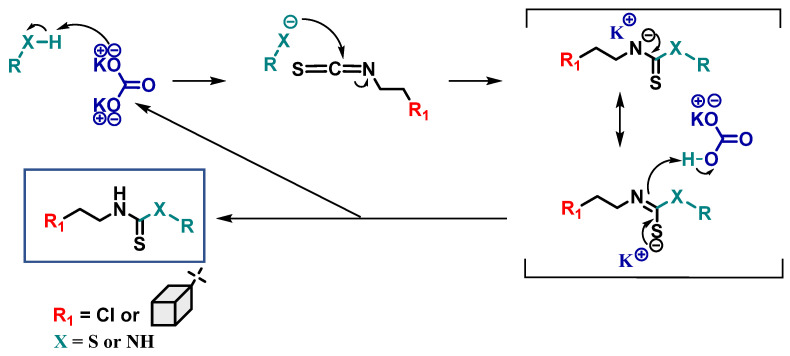
Proposed mechanism of linear product formation.

Similarly to the assumed mechanism of N,S-heterocyclic product formation, we proposed a mechanism of linear S-thiocarbamates formation based on the available literature (Scheme 5).

The conversion occurs in the presence of a nucleophile that is not only responsible for a more electrophilic character of isocyanate but also accelerates the process by deprotonating the thiol molecule, generating the thiolate anion that works as a much stronger nucleophile [46,47,48,49]. Thiolate anion undergoes the addition to the carbon atom of an isocyanate moiety creating an intermediate. The negative charge located on the nitrogen atom is responsible for taking back the hydrogen atom from the potassium carbonate, leading to the linear product and regenerated catalyst.

### 2.5. Thermal Stability

The thermal properties of selected SQ derivatives were investigated with the thermogravimetric analysis in the nitrogen atmosphere. The results are shown in Table 2 (see ESI, Figures S32–S34). The analysis of the obtained thermograms recorded in the N_2_ atmosphere revealed that the incorporation of the SQ moiety into the isocyanates and isothiocyanates strongly influenced the thermal properties of the resulting products (**P1**–**P6**). The 5% mass loss of products was observed at a minimum of 236 °C; meanwhile, the initial decomposition of substrates was already significant at 47 °C for isothiocyanate and at a temperature lower than 30 °C for isocyanates. This result is strictly related to the high stability of the SQ core, because of the high dissociation energy of the Si–O bond, which is much higher than that of typical organic units containing C–C and C–H bonds [50,51]. The final transition of the substrates was completed below 150 °C, while no total mass loss was observed in any of the SQ-functionalized products, even at 1000 °C. The deceptive increase in the residue at 1000 °C compared to that at 300 °C was explained by possible oxidation reactions and the buoyancy effect, according to which, the density of surrounding gas decreases leading to an apparent weight gain. Based on the thermograms, the isothiocyanate derivatives in the form of N,S-heterocycles (**P1**, **P4**) are more thermally stable than the analogous O,N-heterocycles (**P5**, **P6**) or linear products (**P2**, **P3**).

### 2.6. Photophysical Properties

**Figure 3:**
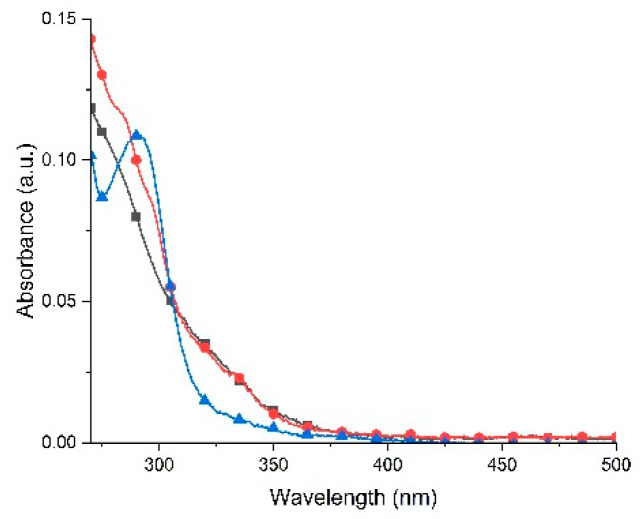
The UV–Vis absorption spectra of P8 (■), P7 (●), and P9 (▲) measured in dichloromethane solutions at room temperature.

The photophysical properties of compounds obtained were investigated in both diluted dichloromethane solution and in the solid state in order to check their potential applications in the area of optics. As expected, compounds **P1**–**P6** showed no absorption in the range of 800–270 nm due to the lack of conjugated systems of π-electrons. For compounds **P7**–**P9,** the UV absorption was observed (Figure 3) and attributed to their π–π* and n–π* transitions. The UV–Vis absorption spectrum of methoxybenzene derivative **P8** shows an absorption band extending from 270 nm with an absorption onset at approximately 360 nm typical of the methoxybenzene group [52]. A similar but slightly more structured absorption profile was observed for the anthracene derivative **P7** [53]. In turn, the spectrum of **P9** showed a red-shifted, distinct UV absorption band with an absorption maximum at 290 nm due to the presence of a more conjugated substitute in its structure [54]. The absorption profiles of **P7**–**P9** were consistent with those observed for **2a**–**c** substrates (see ESI, Figure S35).

**Figure 4:**
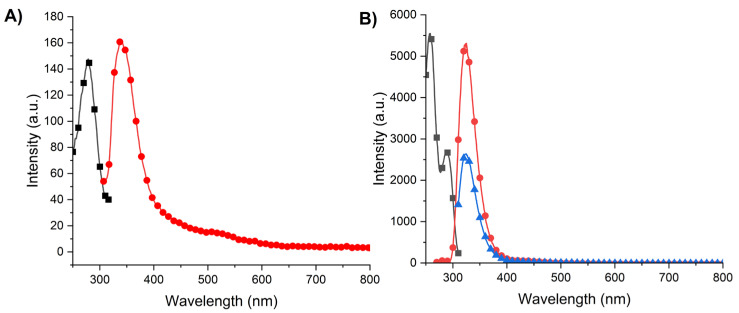
(**A**) Excitation (λem. = 340 nm ■) and emission (λex. = 278 nm ●) spectra of P7 in dichloromethane; (**B**) excitation (λem. = 324 nm; ■) and emission (λex. = 258 nm ● and λex. = 291 nm ▲) spectra of P9 in dichloromethane.

The photoluminescent behavior of the compounds obtained was at first investigated in dilute dichloromethane solutions (Figure 4).

**Figure 5:**
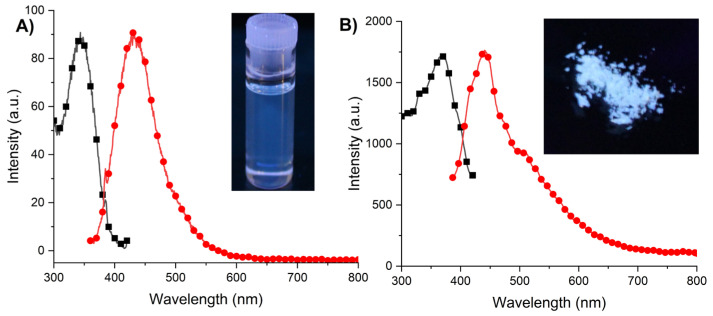
Excitation (■) and emission (●) spectra of P9 in dichloromethane solution (**A**) (λ_em._ = 435 nm and λ_ex._ = 345 nm) and in the solid state (**B**) (λ_em._= 441 nm and λ_ex._ = 368 nm). Insert: The photographs showing the emission of P9 in dichloromethane solution (**A**) and the solid state (**B**) when irradiated with the handheld UV lamp (λ = 365 nm).

The methoxybenzene derivative **P8** did not exhibit any emission. Figure 5 shows the excitation and emission spectra of **P7** and **P9**. The naphthalene derivative **P7** spectrum exhibited one excitation maximum at 278 nm, while that of **P9** exhibited two excitation maxima at 258 nm and 291 nm. When excited at the excitation maxima, **P7** was found to exhibit weak structureless emission in the UV range with a maximum at 340 nm similar to other naphthalene derivatives [55,56]. For **P9**, the excitation at the excitation maxima resulted in a strong emission with the maximum at 324 nm.

Interestingly, when irradiated with the handheld UV lamp (λ = 365 nm), the solution of **P9** exhibited blue emission (Figure 5A insert), which was probably caused by the formation of excimers in the solution and as seen in Figure 5A, additional excitation and emission bands were found for this compound with the maxima at 345 nm and 435 nm, respectively. The bright blue emission was also observed in the solid state (Figure 5B insert). When the compound was excited at the excitation maximum (λ = 368 nm), a broad emission band was observed at 441 nm, extending to about 650 nm (Figure 5B). It was observed that the emission properties of compounds **P7**–**P9** were similar to those observed for corresponding substrates **2a**–**c** (see ESI, Figures S36 and S37). This indicates that the incorporation of the SQ moiety does not modify the photoluminescence properties.

## 3. Materials and Methods

### 3.1. General Methods and Chemicals

Unless indicated otherwise, all operations were carried out under aerobic conditions. ^1^H NMR and ^13^C NMR spectra were recorded at 25 °C in CDCl_3_ on a Varian 400 (Palo Alto, CA, USA) operating at 402.6 and 101.2 MHz, respectively. ^29^Si NMR were recorded on a Brucker Ascend 400 Nanobay (Bellerica, MA, USA) operating at 79.50 MHz. Chemical shifts are reported in ppm with reference to the residual solvent peaks for ^1^H and ^13^C NMR and to TMS for ^29^Si NMR. Thin layer chromatography (TLC) was conducted on plates coated with a 250 μm thick silica gel layer and column chromatography was performed on silica gel 60 (70–230 mesh). ESI-MS spectra were obtained using Synapt Gs-S HDMS (Waters, Milford, MA, USA) mass spectrometer with electrospray ion source and quadrupole time-of-flight analyzer with a resolving power of FWMH 38000. Acetonitrile was used as the sample solvent. The Capillary Voltage was set to 4.5 kV, the sampling was set to 40, and the source temperature was equal to 120 °C. The most abundant ions in the ESI-MS spectra were the protonated ions of desired products. The TGA analyses were performed using a thermogravimetric analyzer TGA4000 (Perkin-Elmer, Waltham, MA, USA). The heating rate was set to 10 °C/min. The analyses were made using nitrogen as carrying gas at a flow rate of 20 mL per minute. The samples (approx. 10 mg) were heated from 0 to 1000 ^°^C. Absorption spectra were recorded with a Jasco V-770 UV–Vis-NIR spectrometer (Tokyo, Japan). Fluorescence measurements were performed with a Jasco FP-8500 spectrometer. The absorption of the solutions of the compounds for fluorescence measurements was around 0.1 at the excitation maximum. The excitation and emission spectra in the solid state were recorded using an integrating sphere (ϕ = 10 cm) that had been calibrated using a calibrated light source.

All reagents that were commercially available and used without further purification, were purchased from the following sources: mercaptopropylisobutyl-POSS (**SQ-SH**), aminopropylisobutyl-POSS (**SQ-NH_2_**) (Hybrid Plastic, Hattiesburg, MS, USA), thiols, isocyanates, isothiocyanates (Chemat, Gdańsk, Poland), potassium carbonate (Merck, Darmstadt, Germany), chloroform-d_1_ (Deutero, Kastellaun, Germany) and acetone, DCM, *n*-hexane (Fisher Chemical, Loughborough, UK). **SQ-NCS** [57], and bulky amine (**2c**) [58] were synthesized using available literature data.

### 3.2. General Procedure for Catalytic Tests

An oven-dried 5 mL glass reactor quipped with a magnetic stirring bar was charged under aerobic conditions with **K_2_CO_3_** (0.6 equiv., 5 mg, 2.16 × 10^−5^ mol), **1a** (1 equiv., 30 mg, 3.36 × 10^−5^ mol) and **2a** (1 equiv., 3.46 µL, 3.36 × 10^−5^ mol). The reaction mixture was stirred at 60 °C for 1–72 h. Next, the solvent was evaporated under vacuum and the residue was analyzed using ^1^H NMR spectroscopy.

### 3.3. General Procedure for the Synthesis of Products **P1–P3**

An oven-dried 5 mL glass reactor quipped with a magnetic stirring bar was charged in aerobic conditions with **SQ-SH** (100 mg, 1.12 × 10^−4^ mol, 1 equiv.), **1a–c** (1.12 × 10^−4^ mol, 1 equiv.) and **K_2_CO_3_** (9.28 mg, 6.72 × 10^−5^ mol, 0.6 equiv.). The reaction mixture was stirred at 60 °C for 5 h. Then, it was filtered through Celite and the evaporation of the solvent afforded analytically pure compounds.

### 3.4. General Procedure for the Synthesis of Products **P4–P6**

The syntheses were carried out according to the procedure described for **P1**–**P3**, using the following amounts of reagents: **SQ**-**NH_2_** (100 mg, 1.14 × 10^−^^4^ mol, 1 equiv.), **1a**–**c** (1.14 × 10^−^^4^ mol, 1 equiv.), and **K_2_CO_3_** (9.45 mg, 6.55 × 10^−^^5^ mol, 0.6 equiv.).

### 3.5. General Procedure for the Synthesis of Products **P7–P9**

The syntheses were carried out according to the procedure described for **P1**–**P3**, using the following amounts of reagents: **SQ**-**NSC** (100 mg, 1.09 × 10^−^^4^ mol, 1 equiv.), **1a**–**c** (1.09 × 10^−^^4^ mol, 1 equiv.), and **K_2_CO_3_** (9.45 mg, 6.55 × 10^−^^5^ mol, 0.6 equiv.).

### 3.6. Synthesis of Product **P1** on a Preparative Scale

A 10 mL high-pressure Schlenk vessel equipped with a magnetic stirring bar was charged under aerobic conditions with **SQ-SH** (500 mg, 5.60 × 10^−4^ mol, 1 equiv.), **1a** (53.89 µL, 5.60 × 10^−4^ mol, 1 equiv.) and **K_2_CO_3_** (46.4 mg, 3.36 × 10^−4^ mol, 0.6 equiv.). The reaction mixture was stirred at 60 °C for 24 h and then it was filtered through Celite. The evaporation of the solvents gave an analytically pure product (white solid, 0.504 g, 5.17 × 10^−4^ mol, 92%).

## 4. Conclusions

In summary, a new base-catalyzed protocol for the synthesis of novel classes of functionalized SQs was designed and optimized. For all combinations of used reagents, the reactions proceeded effectively leading to the exclusive formation of heterocyclic or linear products. Among the most important advantages of the designed methodology we can distinguish, there was no need to use transition metal catalysts, the use of green solvents, elimination of any other additives, simple isolation process, and full chemoselectivity. All syntheses can be conducted under air conditions at relatively low temperatures, which is fully consistent with the green chemistry rules. The reaction can be easily scalable to the gram quantities offering substantial benefits for practical applications. Nine new modified SQs were synthesized and characterized by spectroscopic and spectrometric methods. The thermal analyses conducted for substrates and products **P1**–**P6** proved that the implementation of the silsesquioxyl group has a significant impact on the thermal properties of the final materials. Products **P7**–**P9** were also verified in terms of their absorption and emission properties. We showed that **P7**, **P8** are luminescent with the maximum emission in the ultraviolet range. The most interesting luminescence properties were exhibited by **P9,** which has multiple aromatic rings within its structure. It exhibits bright blue emission in both the solution and solid state; therefore, it could find application as the blue light-emitting material. It is especially interesting in view of the fact that there were some reports about using such light in the cancer therapies [59,60], OLED manufacturing [61], microbial inactivation [62], and food product preservation [63].

## Figures and Tables

**Table 1:** Functionalization of **SQ-SH** with **1a**. Optimization of the reaction conditions.

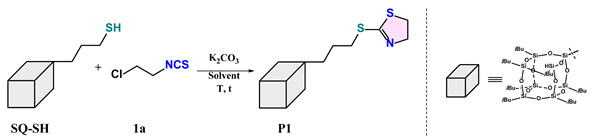						
Entry	Solvent	Temp. [°C]	K_2_CO_3_ [equiv.]	Time [h]	Aerobic Conditions	Conv. of 1a ^[a]^ [%]
1	Toluene	120	0.6	24	-	85
2	Toluene	100	0.6	24	-	95
3	Toluene	80	0.6	24	-	95
4	Toluene	60	0.6	24	-	100
5	Toluene	40	0.6	48	-	80
6	Toluene	25	0.6	72	-	62
7	Toluene	60	0.6	24	+	100
8	Acetone	60	0.6	12	+	100
9	Acetone	40	0.6	48	+	78
10	Acetone	60	0.3	24	+	75
11	Acetone	60	0.1	48	+	42
**12**	**Acetone**	**60**	**0.6**	**5**	**+**	**100**
13	Acetone	60	0.6	2	+	36
14	Acetone	60	0.6	1	+	11
15	Acetone	60	-	72	+	0
16	iPrOH	60	0.6	5	+	92
17	MIBK	60	0.6	5	+	100
18	EtOAc	60	0.6	5	+	10

Reaction condition: Solvent, [**SQ-SH**]:[**1a**]:[**K_2_CO_3_**] = 1:1:0.6. ^[a]^ Determined by ^1^H NMR analysis.

**Table 2:** Thermal properties of pure isocyanate (**1a**–**c**) and SQ-containing products (**P1**–**P6**).

Entry	Compound	Temp. of Mass Lost [°C]		Residue [%]	
		5%	50%	300 °C	1000 °C
1	1a	47	127	1.2	1.4
2	1b	28	79	1.6	2.0
3	1c	29	88	2.8	2.6
4	P1	290	342	92	13
5	P2	236	333	74	16
6	P3	256	336	74	20
7	P4	291	362	93	15
8	P5	269	344	86	11
9	P6	274	353	88	21

Measurements made in N_2_ atmosphere.

## Data Availability

All data generated or analyzed during this study are included in this published article (and its Supplementary Information files: Analytical data of isolated products and catalyst, NMR spectra of isolated products: Figures S1–S78, XRD analysis: Table S1, Figure S79).

## Supplementary Materials

The following supporting information can be downloaded at https://www.mdpi.com/article/10.3390/ijms26167769/s1.
