# Supplementary material for: Base-Catalyzed Pathway Towards Isocyanate Derivatives of Silsesquioxanes
Source: Int J Mol Sci. 2025 Aug 12;26(16):7769. doi: 10.3390/ijms26167769 (PMC12386852; doi:10.3390/ijms26167769)
Supplement: Supplementary file 1 [file ijms-26-07769-s001.zip › ijms-3784733-supplementary.pdf]

Supporting Information

For

Base-catalyzed pathway towards isocyanate derivatives of silsesquioxanes

Kamil Hanek, Monika Wałęsa-Chorab and Patrycja Żak*

Faculty of Chemistry, Adam Mickiewicz University in Poznan, Uniwersytetu Poznańskiego 8, 61-614 Poznań, Poland. E-mail: pkw@amu.edu.pl

CONTENTS:

**1.**	**Analytical data of substrates 2c and SQ-NCS**	**S-2**
**2.**	**Analytical data of products P1-P9**	**S-2**
**3.**	**NMR spectra of substrates 2c and SQ-NCS**	**S-6**
**4.**	**NMR spectra of products P1-P9**	**S-8**
**5.**	**Thermogravimetric analyses**	**S-21**
**6.**	**UV-VIS analyses of substrates**	**S-22**
**7.**	**References**	**S-24**

1. Analytical data of substrates 2c and SQ-NCS

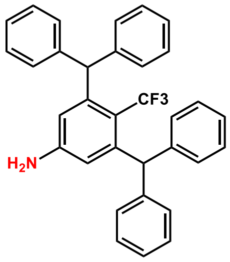

2c

White solid, isolated yield: 92%; ^{1}H NMR (400 MHz, $CDCl_3$, 296K): δ (ppm) 3.75 (s, 2H, NH_2), 5.40 (s, 2H, C$H$$Ph_2$), 6.83 (s, 2H, m-$CH_{Ar}$), 7.03-7.10 (m, 8H, C_6H_5), 7.20-7.25 (m, 4H, C_6H_5), 7.27-7.34 (m, 8H, C_6H_5); ^{13}C NMR (100 MHz, $CDCl_3$, 296K): δ (ppm) 52.54 ($CHPh_2$), 119.22 (q, J = 32.1 Hz), 123.50, 125.49 (q, J = 3.8 Hz), 126.20, 128.69, 128.77, 129.36, 141.58, 145.21; MS (ESI+): m/z 494 $[M+H]^+$.

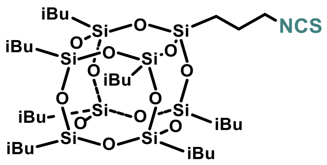

SQ-NCS

White solid, isolated yield: 98%; ^{1}H NMR (400 MHz, $CDCl_3$, 296K): δ (ppm) 0.54 (dd, 14H, J_{HH} = 7.0, 3.2 Hz, CH_2), 0.59 – 0.66 (m, 2H, CH_2), 0.89 (dd, 42H, J_{HH} = 6.6, 1.9 Hz, CH_3), 1.70-1.86 (m, 9H, CH_2 and CH), 3.43 (t, 2H, J_{HH} = 6.7 Hz, NCH_2); ^{13}C NMR (100 MHz, $CDCl_3$, 296K): δ (ppm) 9.37 (CH_2), 22.44 (CH_2), 22.48 (CH_2), 23.83 (CH), 23.89 (CH), 24.03 (CH_2), 25.66 (CH_3), 25.67 (CH_3), 47.22 (NCH_2), 164.65 (NCS); MS (ESI+): m/z 938 $[M+Na]^+$. These NMR data matched those reported in the literature.[S1]

2. Analytical data of products P1-P12

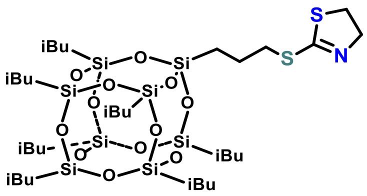

Product P1

White solid, isolated yield: 97%; ^{1}H NMR (400 MHz, $CDCl_3$, 296K): δ (ppm) 0.60 (dd, 14H, J_{HH} = 7.1, 2.0 Hz, CH_2), 0.70 – 0.75 (m, 2H, CH_2), 0.88 – 0.92 (m, 42H, CH_3), 1.78 – 1.90 (m, 9H, CH_2 and CH), 3.10 (t, 2H, J_{HH} = 7.3 Hz, CH_2), 3.36 (t, 2H, J_{HH} = 8.0 Hz, SCH_2), 4.20 (t, 2H, J_{HH} = 8.0 Hz, SCH_2); ^{13}C NMR (100 MHz, $CDCl_3$, 296K): δ (ppm) 11.57 (CH_2), 22.44 (CH_2), 22.51 (CH_2), 23.00 (CH_2), 23.83 (CH_2), 23.87 (CH), 23.88 (CH), 25.67 (CH_3), 25.69 (CH_3), 34.27 (CH_2), 35.50 (NCH_2), 64.35 (SCH_2), 165.60 (NCS); ^{29}Si NMR (100 MHz, $CDCl_3$, 296K): δ (ppm) -67.63, -67.90, -68.20; MS (ESI+): m/z 976 $[M+H]^+$.

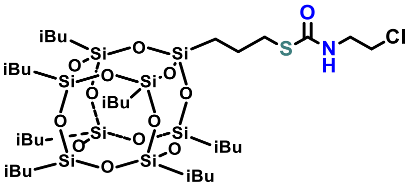 **Product P2**	White solid, isolated yield: 98%; ^{1}H NMR (400 MHz, $CDCl_3$, 296K): δ (ppm) 0.55 – 0.64 (m, 14H, CH_2), 0.68 – 0.73 (m, 2H, CH_2), 0.89 – 1.01 (m, 42H, CH_3), 1.67 – 1.74 (m, 2H, CH_2), 1.80 – 1.90 (m, 7H, CH_2), 2.48– 2.57 (m, 1H, CH_2), 2.93 (t, 2H, J_{HH} = 7.1 Hz, CH_2), 3.61 – 3.63 (m, 2H, CH_2), 5.73 (br s, 1H, NH); ^{13}C NMR (100 MHz, $CDCl_3$, 296K): δ (ppm) 11.45 (CH_2), 22.40 (CH_2), 22.47 (CH_2), 23.83 (CH), 23.86 (CH), 25.67 (br s, CH_3), 27.37 (CH_2), 27.65 (CH_2), 32.83 (CH_2), 43.67 (SCH_2), 167.83 (NCS); ^{29}Si NMR (100 MHz, $CDCl_3$, 296K): δ (ppm) -67.64, -67.90, -68.09; MS (ESI+): m/z 1020 $[M+K]^+$.
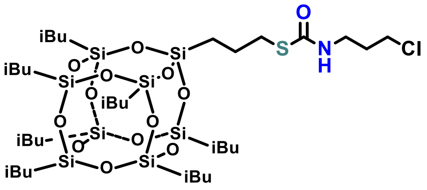 **Product P3**	White solid, isolated yield: 96%; ^{1}H NMR (400 MHz, $CDCl_3$, 296K): δ (ppm) 0.53 – 0.65 (m, 14H, CH_2), 0.67 – 0.73 (m, 2H, CH_2), 0.88 – 1.03 (m, 42H, CH_3), 1.66 – 1.74 (m, 2H, CH_2), 1.79 – 1.89 (m, 7H, CH_2), 1.96 – 2.04 (m, 2H, CH_2), 2.48 – 2.56 (m, 1H, CH_2), 2.92 (t, 1H, J_{HH} = 7.1 Hz, CH_2), 3.35 (dd, 1H, J_{HH} = 12.6, 6.4 Hz, SCH_2), 3.45 (dd, 1H, J_{HH} = 12.6, 6.4 Hz, SCH_2), 3.55 – 3.63 (m, 2H, CH_2), 5.48 (br s, 1H, NH); ^{13}C NMR (100 MHz, $CDCl_3$, 296K): δ (ppm) 11.49 (CH_2), 22.44 (CH_2), 22.51 (CH_2), 23.84 (CH), 23.87 (CH), 25.66 (CH_3), 25.68 (CH_3), 32.23 (CH_2), 32.76 (CH_2), 37.84 (CH_2), 42.15 (CH_2), 42.55 (CH_2), 158.08 (NCS); ^{29}Si NMR (100 MHz, $CDCl_3$, 296K): δ (ppm) -67.65, -67.90, -68.06; MS (ESI+): m/z 1034 $[M+Na]^+$.
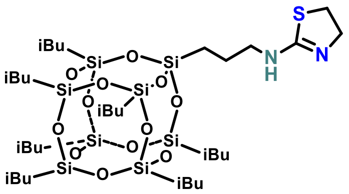 **Product P4**	White solid, isolated yield: 97%; ^{1}H NMR (400 MHz, $CDCl_3$, 296K): δ (ppm) 0.56 – 0.65 (m, 16H, CH_2), 0.90 – 0.99 (m, 42H, CH_3), 1.67 – 1.74 (m, 2H, CH_2), 1.80 – 1.89 (m, 7H, CH_2), 3.27 (t, 2H, J_{HH} = 7.3 Hz, CH_2), 3.38 (t, 2H, J_{HH} = 7.4 Hz, CH_2), 3.98 (t, 2H, J_{HH} = 7.4 Hz, CH_2), 5.67 (br s, 1H, NH); ^{13}C NMR (100 MHz, CD_2Cl_2, 296K): δ (ppm) 10.08 (CH_2), 22.50 (CH_2), 22.54 (CH_2), 23.83 (CH), 23.87 (CH), 24.30 (CH_2), 25.68 (CH_3), 26.59 (CH_2), 33.60 (CH_2), 50.57 (NCH_2), 57.56 (SCH_2), 158.26 (NCS); ^{29}Si NMR (100 MHz, $CDCl_3$, 296K): δ (ppm) -67.62, -67.89 (br s); MS (ESI+): m/z 959 $[M+Na]^+$.

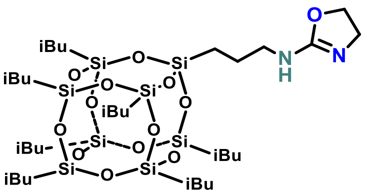 **Product P5**	White solid, isolated yield: 96%; ^{1}H NMR (400 MHz, CD_2Cl_2, 296K): δ (ppm) 0.58 – 0.61 (m, 16H, CH_2), 0.94 – 0.96 (m, 42H, CH_3), 1.56 – 1.65 (m, 2H, CH_2), 1.82 – 1.88 (m, 7H, CH_2), 3.13 (t, 2H, J_{HH} = 7.0 Hz, CH_2), 3.69 (t, 2H, J_{HH} = 8.5 Hz, CH_2), 4.18 (t, 2H, J_{HH} = 8.5 Hz, CH_2); ^{13}C NMR (100 MHz, $CDCl_3$, 296K): δ (ppm) 9.37 (CH_2), 22.42 (CH_2), 22.45 (CH_2), 23.51 (CH_2), 23.47 (CH_2), 23.83 (CH), 23.87 (CH), 25.68 (CH_3), 42.13 (CH_2), 42.96 (CH_2), 45.15 (CH_2), 157.56 (NCN); ^{29}Si NMR (100 MHz, $CDCl_3$, 296K): δ (ppm) -67.62, -67.86 (br s); MS (ESI+): m/z 943 $[M+H]^+$.
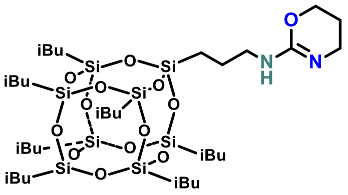 **Product P6**	White solid, isolated yield: 96%; ^{1}H NMR (400 MHz, CD_2Cl_2, 296K): δ (ppm) 0.58 – 0.62 (m, 16H, CH_2), 0.94 – 0.96 (m, 42H, CH_3), 1.50 – 1.61 (m, 2H, CH_2), 1.82 – 1.88 (m, 7H, CH_2), 1.94 (t, 2H, J_{HH} = 6.5 Hz, CH_2), 3.06 – 3.13 (m, 2H, CH_2), 3.26 – 3.30 (m, 2H, CH_2), 3.59 (t, 2H, J_{HH} = 6.5 Hz, CH_2), 4.53 – 4.64 (m, 1H, NH); ^{13}C NMR (100 MHz, $CDCl_3$, 296K): δ (ppm) 9.40 (CH_2), 22.43 (CH_2), 22.46 (CH_2), 23.51 (CH_2), 23.83 (CH), 23.87 (CH), 25.66 (CH_3), 32.78 (CH_2), 32.62 (CH_2), 42.59 (CH_2), 65.60 (CH_2), 157.97 (NCN); ^{29}Si NMR (100 MHz, $CDCl_3$, 296K): δ (ppm) -67.63, -67.86, -67.91; MS (ESI+): m/z 957 $[M+H]^+$.
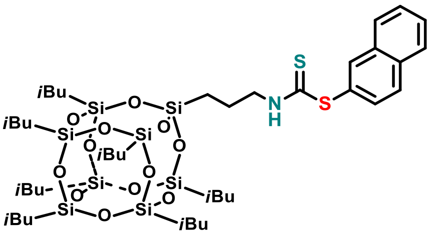 **Product P7**	White solid, isolated yield: 97%; ^{1}H NMR (400 MHz, $CDCl_3$, 296K): δ (ppm) 0.57–0.66 (m, 14H, CH_2), 0.68 – 0.74 (m, 2H, CH_2), 0.95–1.00 (m, 42H, CH_3), 1.80–1.90 (m, 9H, CH_2), 3.50 (t, 2H, J_{HH} = 6.7 Hz, CH_2), 7.14–7.24 (m, 1H, NH), 7.43–7.48 (m, 2H, Ph), 7.63 (dd, 1H, J_{HH} = 8.7, 1.9 Hz, Ph), 7.72–7.75 (m, 1H, Ph), 7.78-7.81 (m, 2H, Ph), 7.99 (d, 1H, J_{HH} = 1.9 Hz, Ph); ^{13}C NMR (100 MHz, CD_2Cl_2, 296K): δ (ppm) 9.37 (CH_2), 22.44 (CH_2), 22.49 (CH_2), 23.84 (CH), 23.90 (CH), 24.05 (CH_2), 25.66 (CH_3), 25.58 (CH_2), 47.21 (NCH_2), 125.69, 126.22, 126.60, 126.71, 127.45, 127.75, 128.95, 132.52, 133.49, 134.29, 164.65 (NCS); ^{29}Si NMR (100 MHz, $CDCl_3$, 296K): δ (ppm) -67.50, -67.83. -68.55.

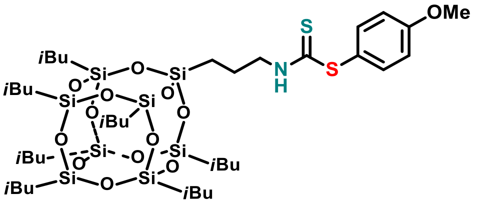 **Product P8**	White solid, isolated yield: 96%; ^{1}H NMR (400 MHz, $CDCl_3$, 296K): δ (ppm) 0.59–0.63 (m, 14H, CH_2), 0.68 – 0.72 (m, 2H, CH_2), 0.94–0.98 (m, 42H, CH_3), 1.79–1.88 (m, 9H, CH_2), 3.80 (t, 2H, J_{HH} = 8.9 Hz, CH_2), 5.23 (s, 1H, N*H*), 6.83 (d, 2H, J_{HH} = 8.9 Hz, C_6H_4-OCH_3), 7.39 (d, 2H, J_{HH} = 8.9 Hz, C_6H_4- OCH_3); ^{13}C NMR (100 MHz, $CDCl_3$, 296K): δ (ppm) 9.36 (CH_2), 22.43 (CH_2), 22.48 (CH_2), 23.83 (*C*H), 23.89 (*C*H), 24.03 (CH_2), 25.65 (CH_3), 25.68 (CH_2), 47.21 (NCH_2), 55.34 (OCH_3), 114.61, 128.46, 129.94, 132.64, 159.92 (N*C*S); ^{29}Si NMR (100 MHz, $CDCl_3$, 296K): δ (ppm) -67.52, -67.85, -68.57.
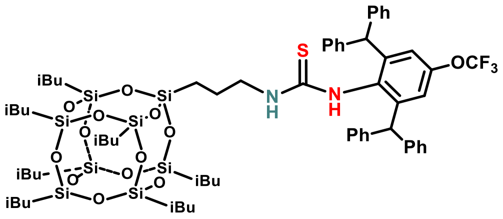 **Product P9**	White solid, isolated yield: 96%; ^{1}H NMR (400 MHz, $CDCl_3$, 296K): δ (ppm) 0.59–0.67 (m, 14H, CH_2), 0.70 – 0.74 (m, 2H, CH_2), 0.93–1.03 (m, 42H, CH_3), 1.80–1.92 (m, 9H, CH_2), 3.51 (t, 2H, J_{HH} = 6.7 Hz, CH_2), 5.42 (s, 2H, C*H*Ph), 6.84 (s, 2H, m-CH_{Ar}), 7.00-7.12 (m, 9H, Ph + N*H*), 7.13-7.25 (m, 5H, Ph + N*H*), 7.27-7.35 (m, 8H); ^{13}C NMR (100 MHz, $CDCl_3$, 296K): δ (ppm) 9.36 (CH_2), 22.43 (CH_2), 22.48 (CH_2), 23.83 (*C*H), 23.89 (*C*H), 24.04 (CH_2), 25.65 (CH_3), 25.68 (CH_2), 47.20 (NCH_2), 52.41 ($CHPh_2$), 125.48, 127.02, 127.86, 128.36, 128.71, 129.31, 141.56, 145.18, 173.20 (N*C*S); ^{29}Si NMR (100 MHz, $CDCl_3$, 296K): δ (ppm) -67.50, -67.76, -67.95.

3. NMR spectra of substrates 2c and SQ-NCS

2c

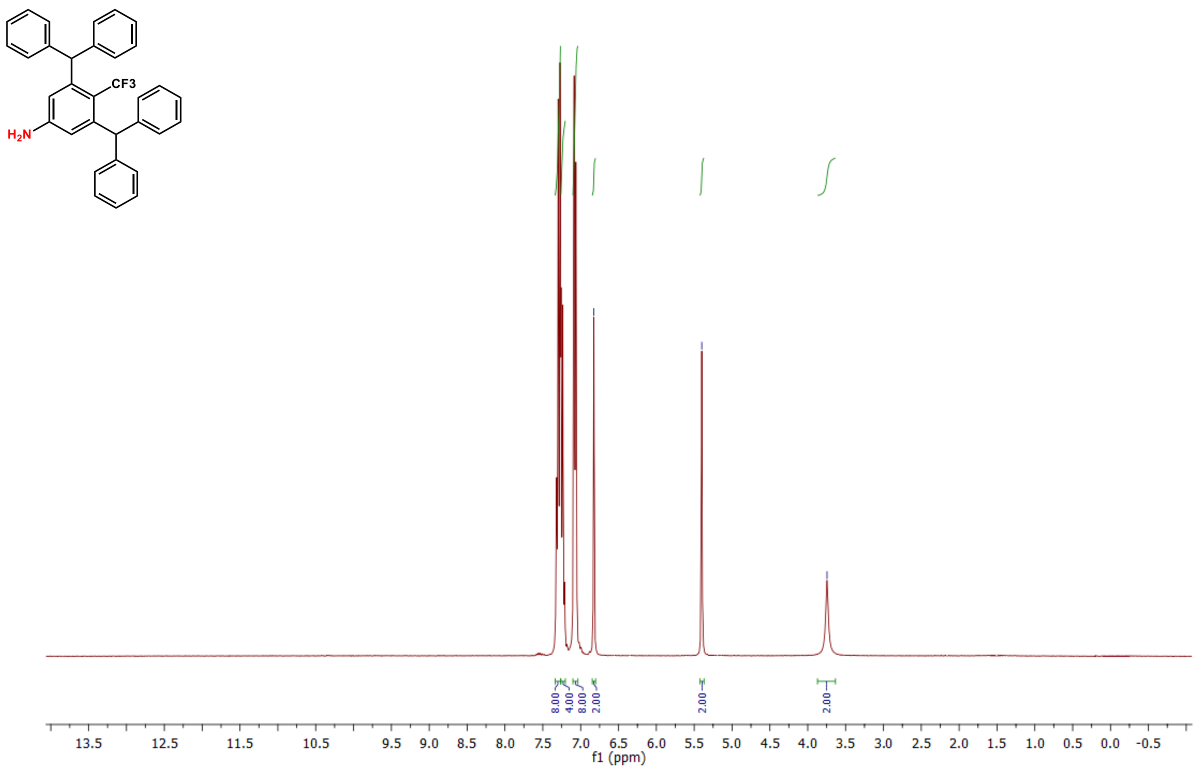

Figure S1. ^{1}H NMR (400 MHz, $CDCl_3$) of substrate **2c**

2c

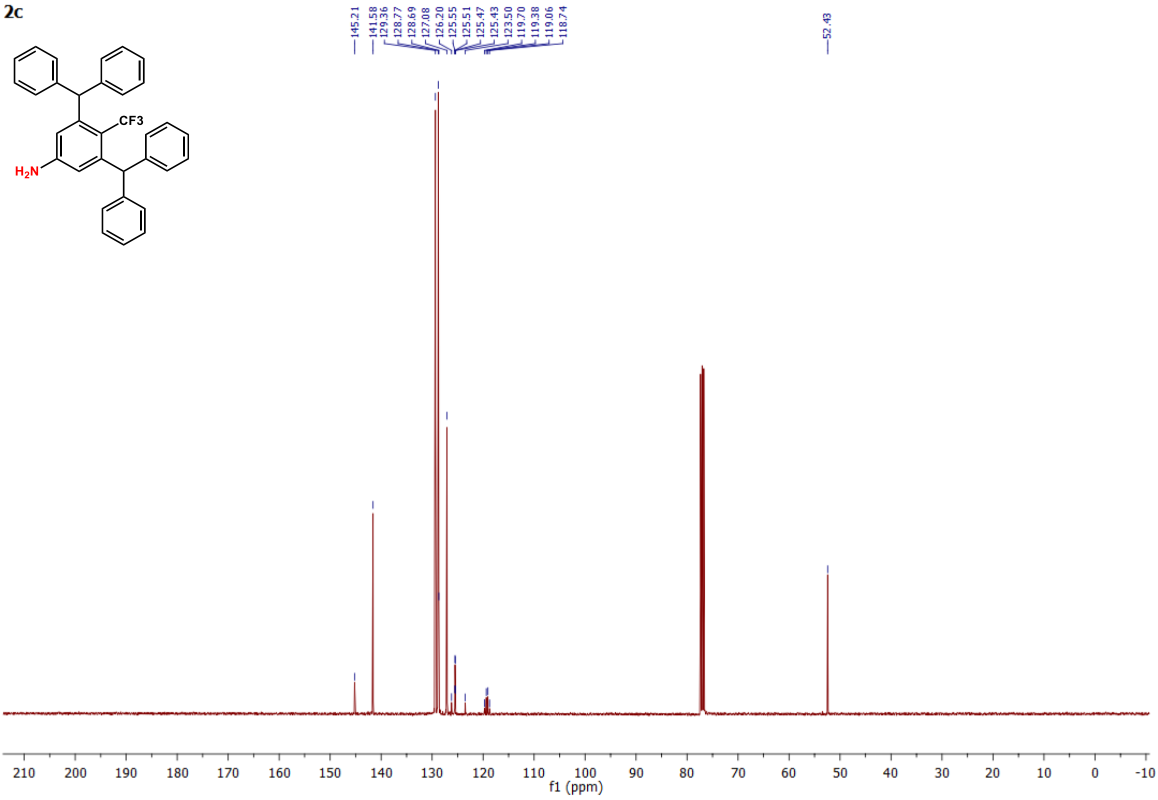

Figure S2. ^{13}C NMR (101 MHz, $CDCl_3$) of substrate **2c**

SQ-NCS

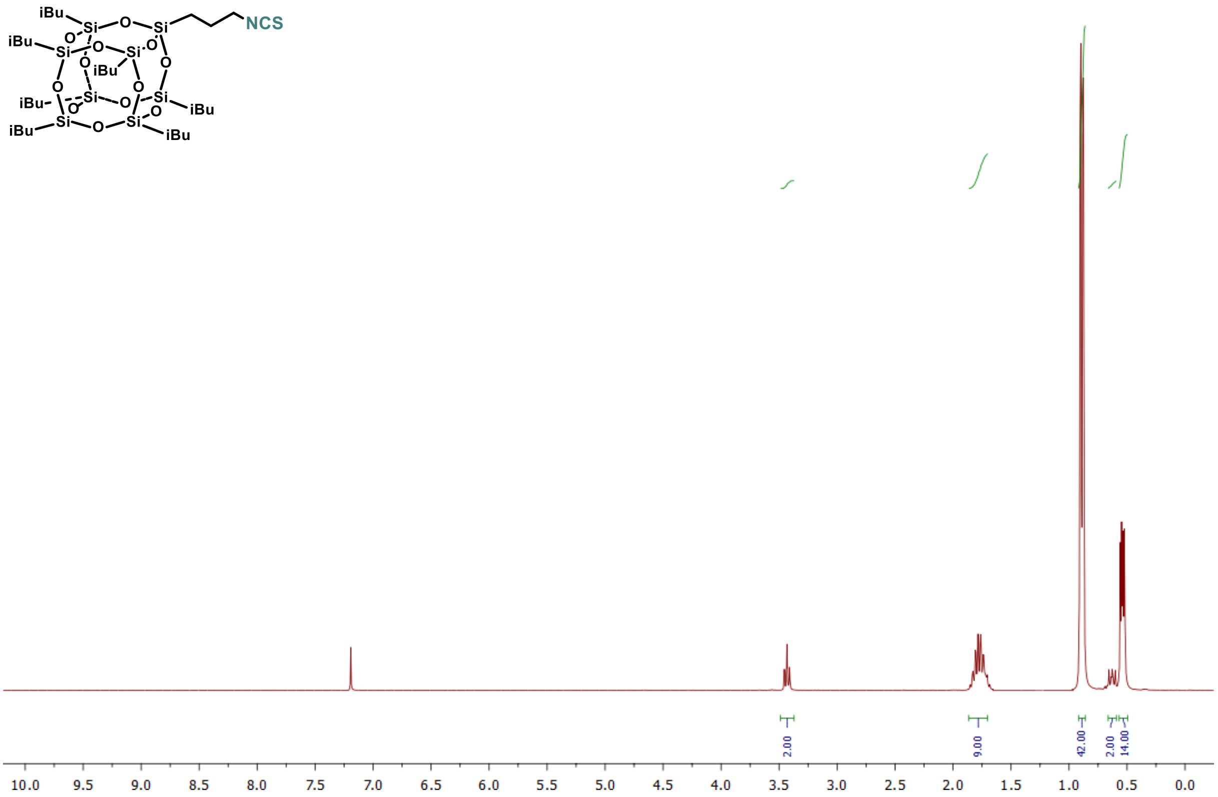

Figure S3. ^{1}H NMR (400 MHz, $CDCl_3$) of substrate **SQ-NCS**

SQ-NCS

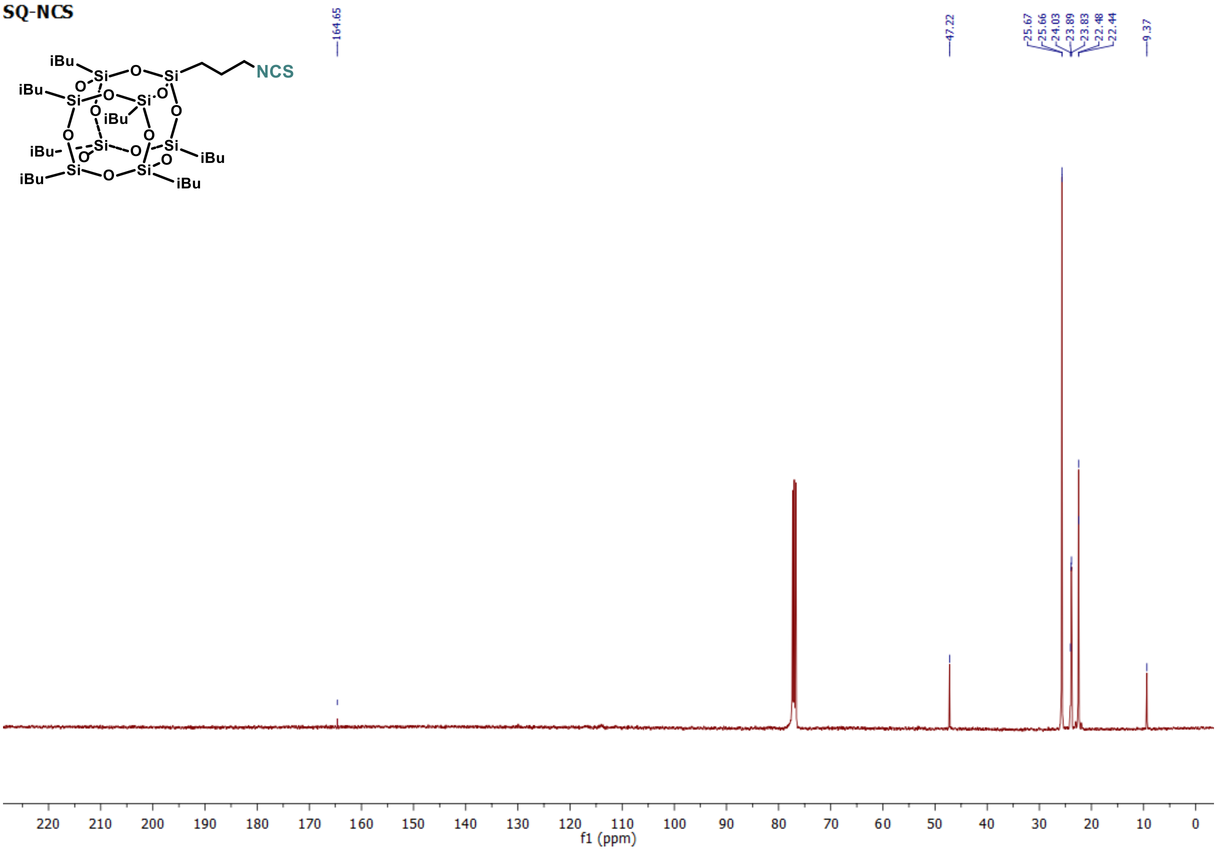

Figure S4. ^{13}C NMR (101 MHz, $CDCl_3$) of substrate **SQ-NCS**

4. NMR spectra of products P1-P9

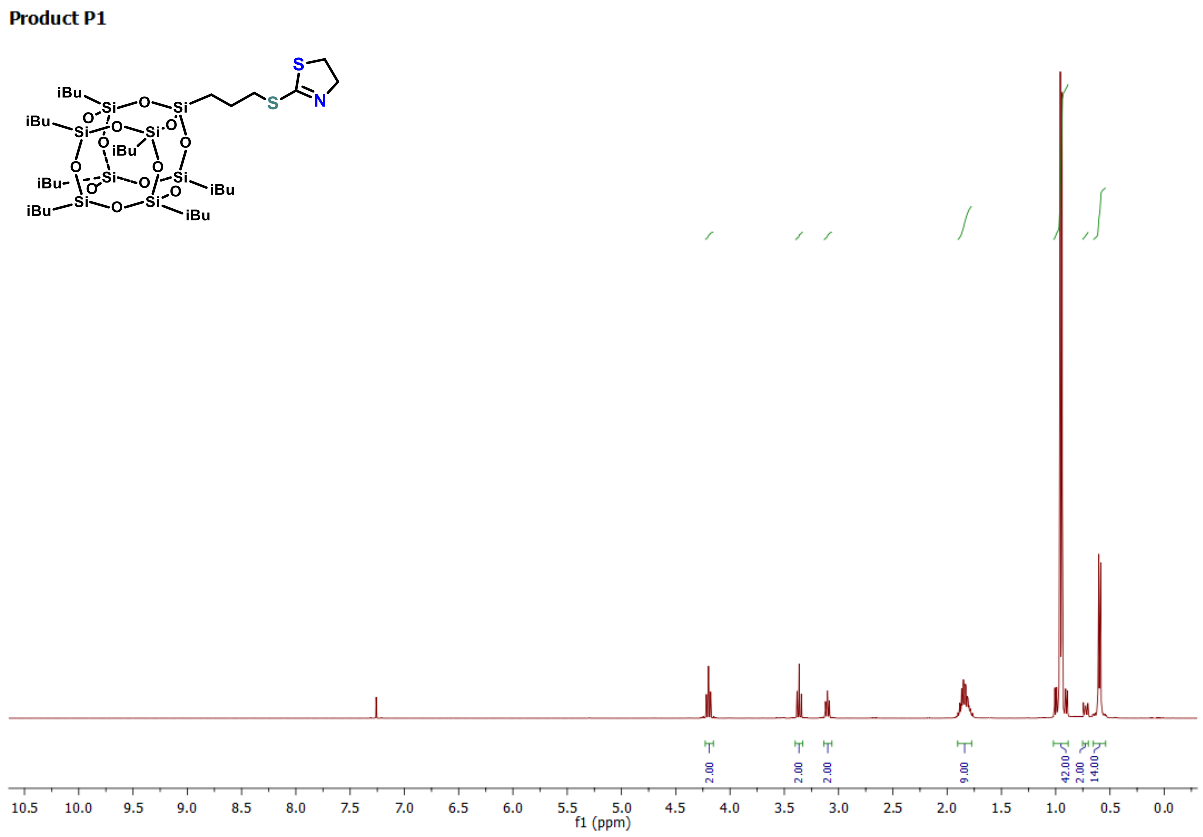

Figure S5. ^{1}H NMR (400 MHz, $CDCl_3$) of product **P1**

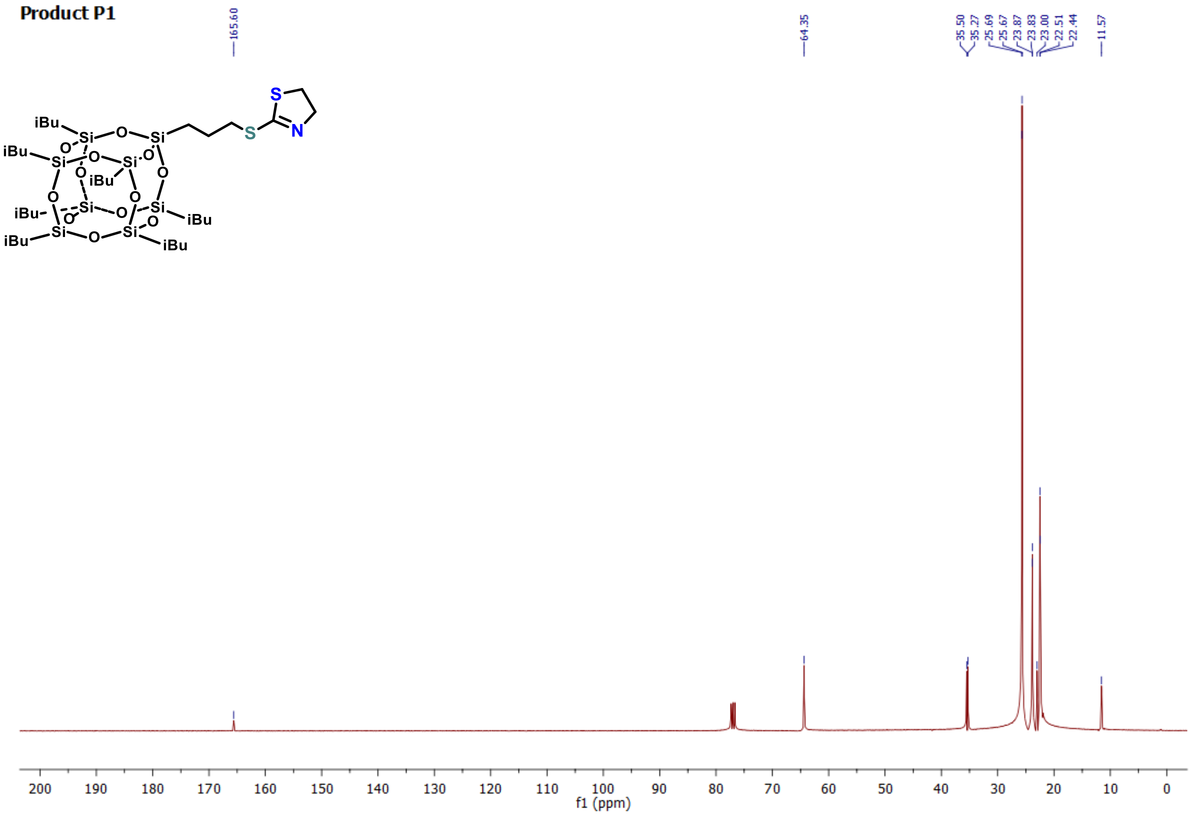

Figure S6. ^{13}C NMR (101 MHz, $CDCl_3$) of product **P1**

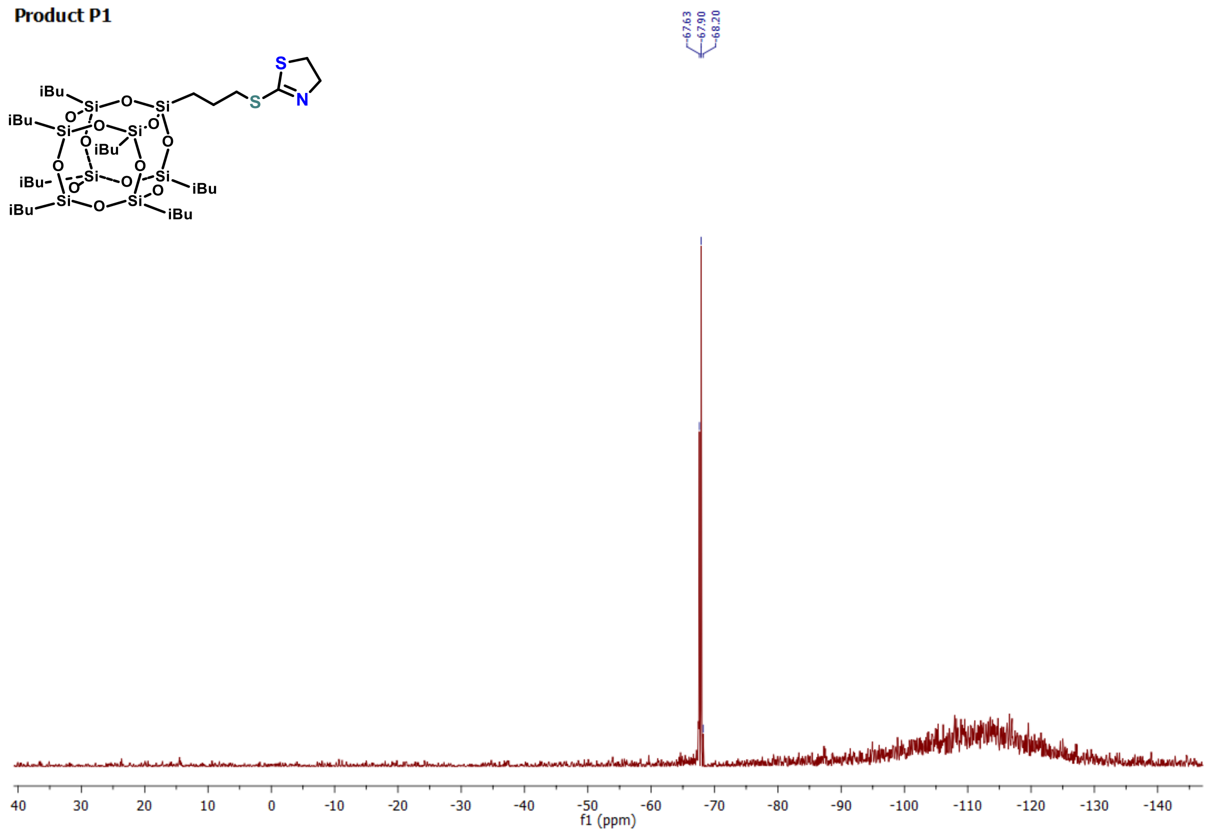

Figure S7. ^{29}Si NMR (79 MHz, $CDCl_3$) of product **P1**

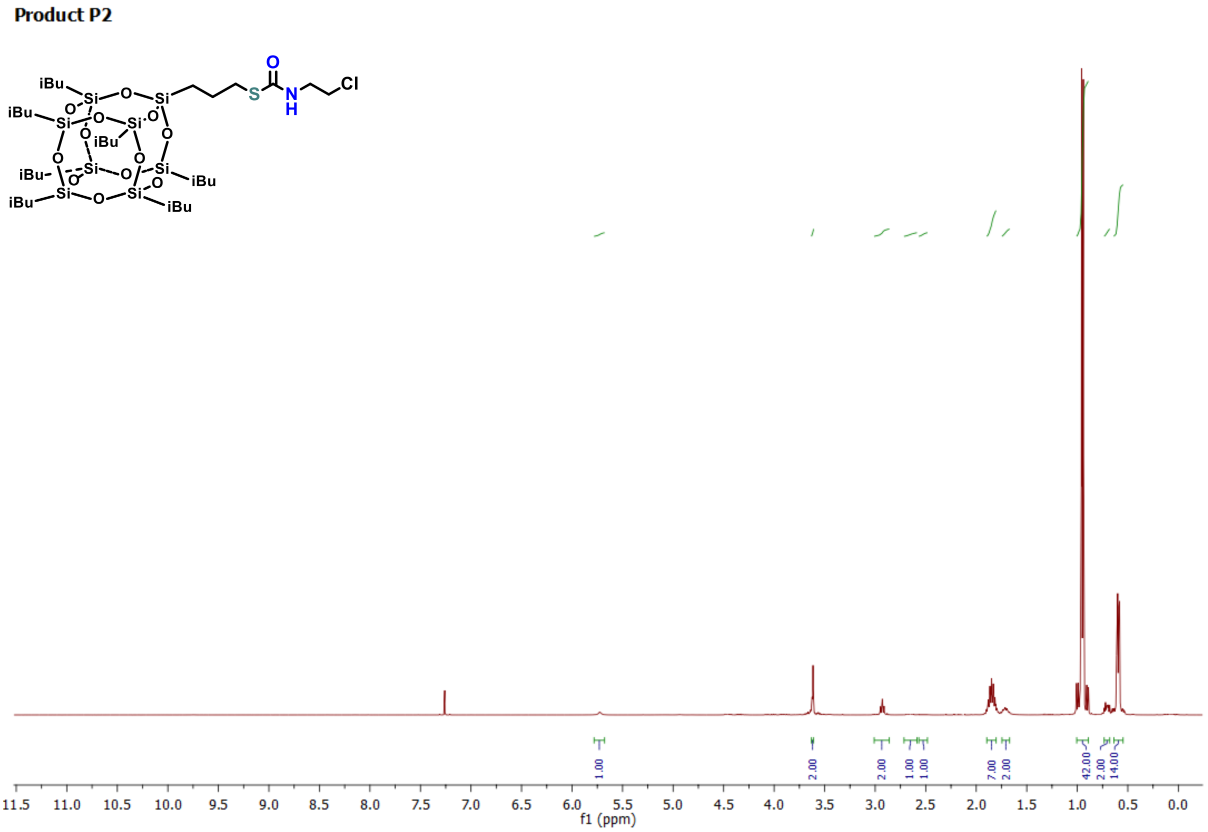

Figure S8. ^{1}H NMR (400 MHz, $CDCl_3$) of product **P2**

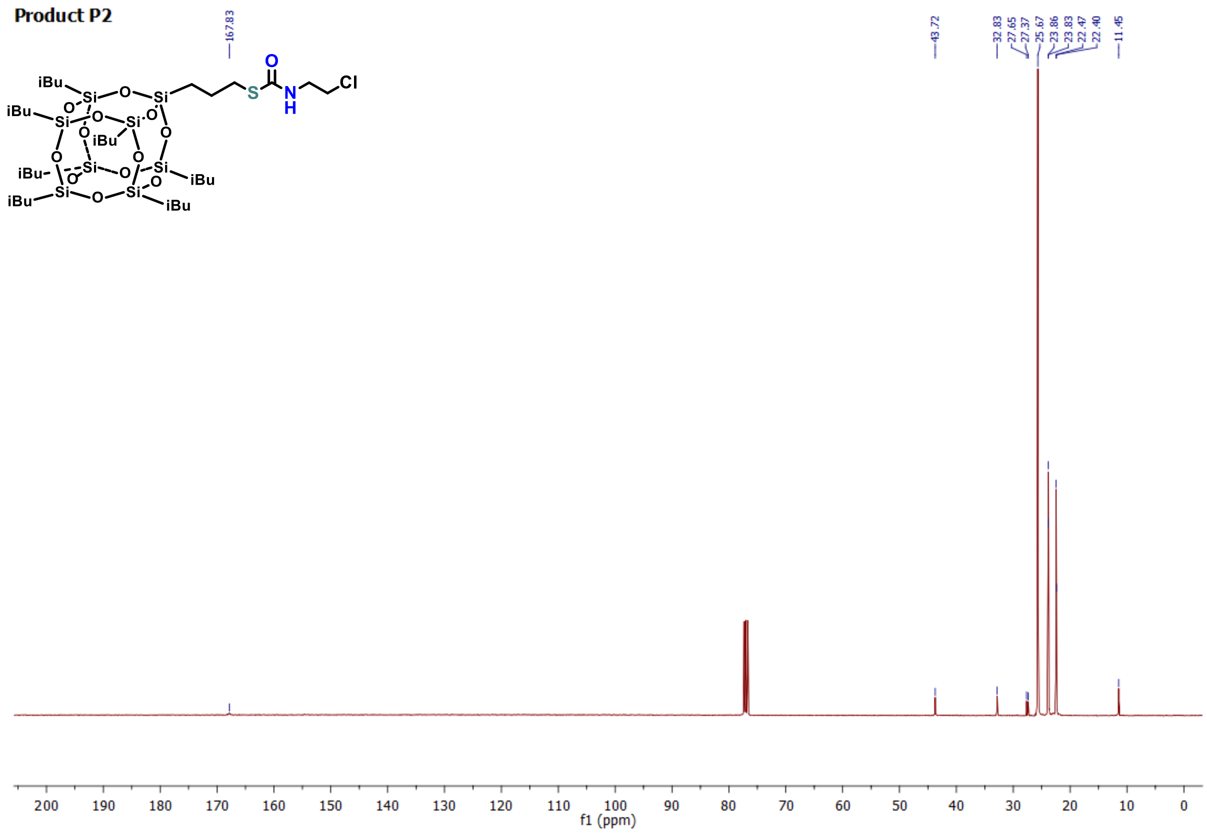

Figure S9. ^{13}C NMR (101 MHz, $CDCl_3$) of product **P2**

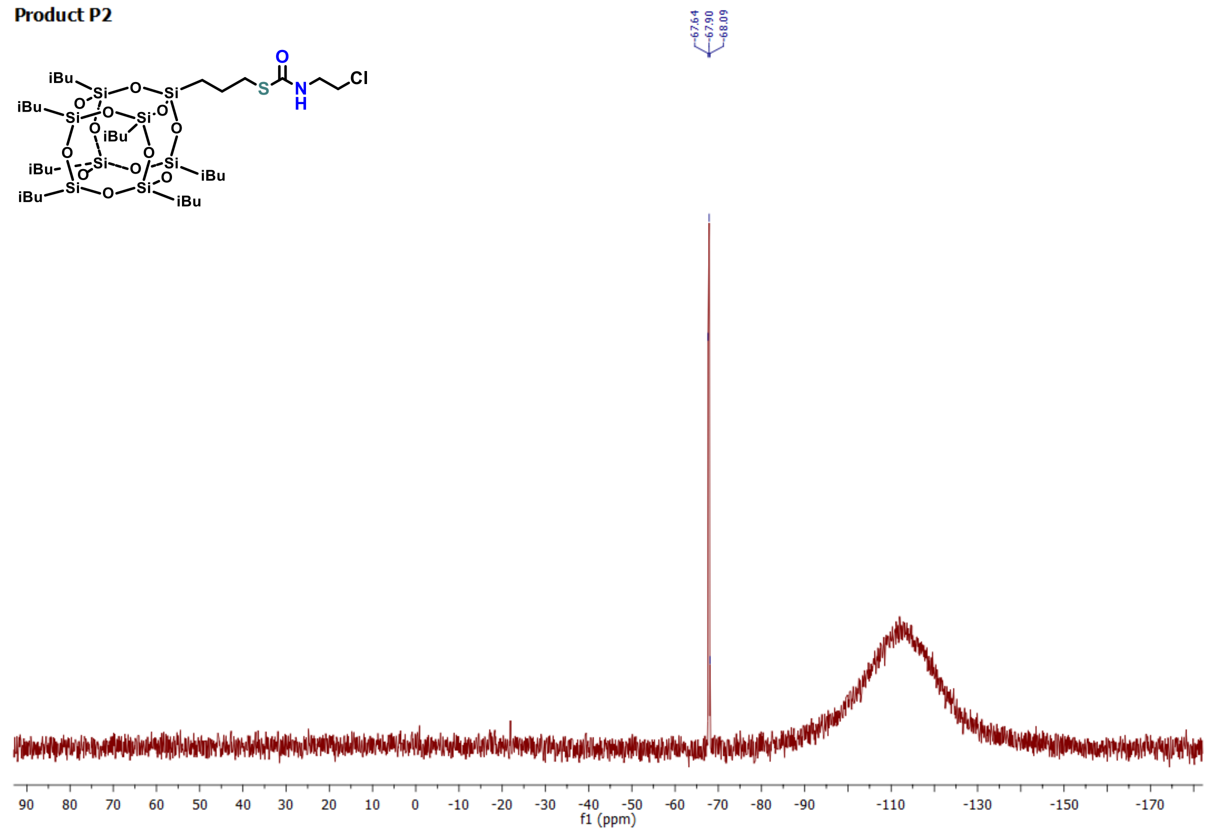

Figure S10. ^{29}Si NMR (79 MHz, $CDCl_3$) of product **P2**

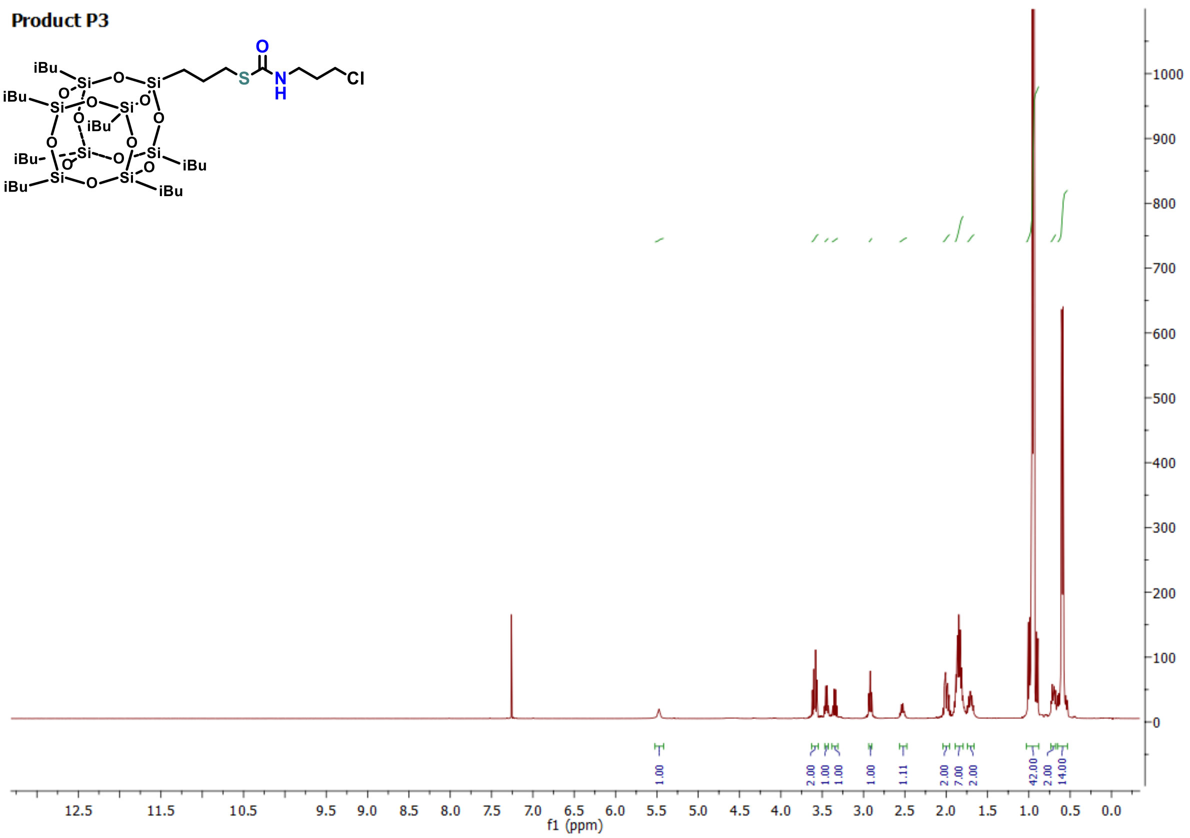

Figure S11. ^{1}H NMR (400 MHz, $CDCl_3$) of product **P3**

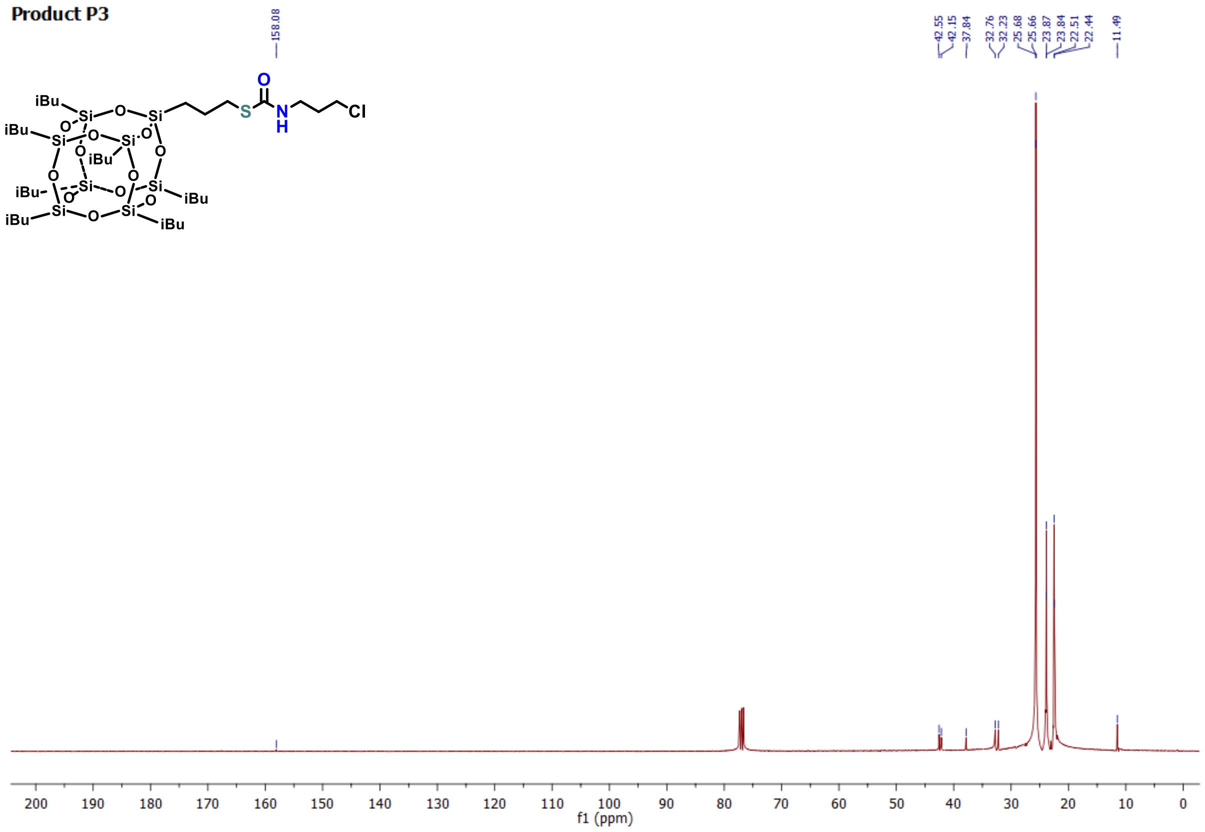

Figure S12. ^{13}C NMR (101 MHz, $CDCl_3$) of product **P3**

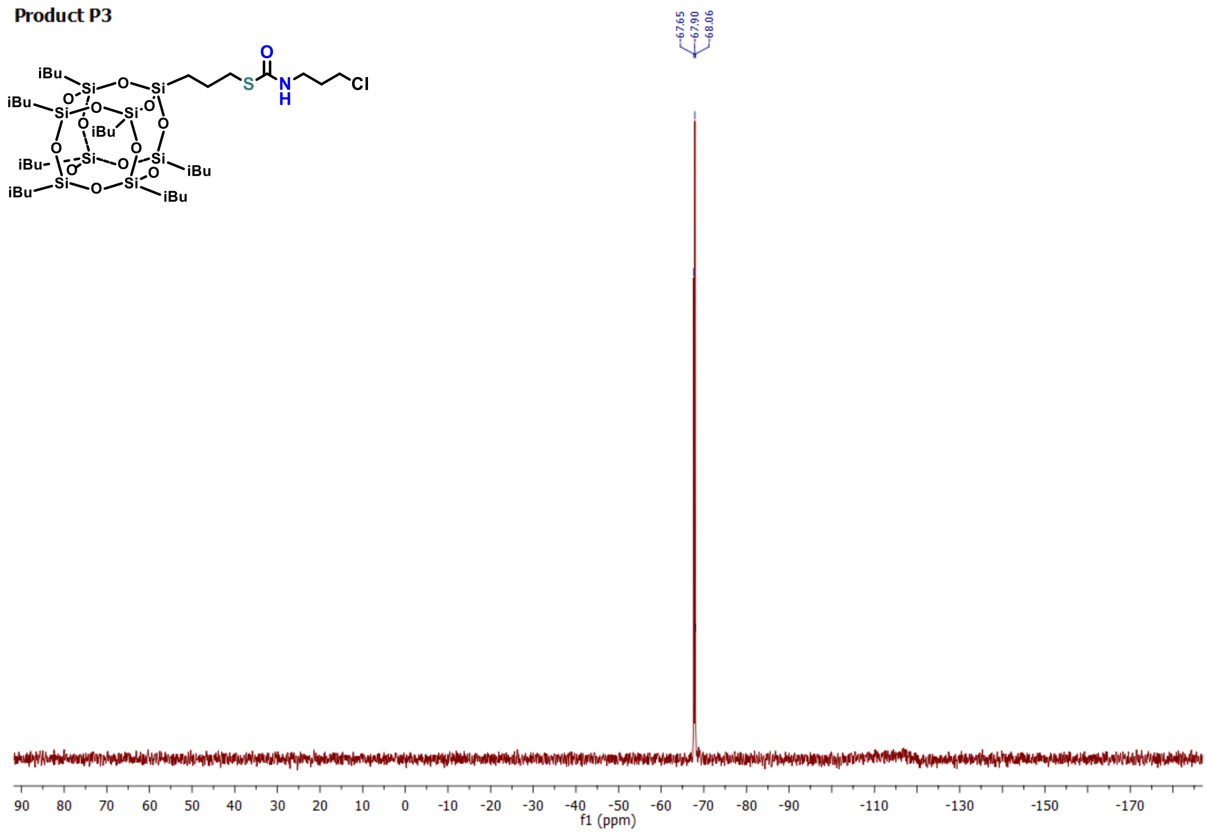

Figure S13. ^{29}Si NMR (79 MHz, $CDCl_3$) of product **P3**

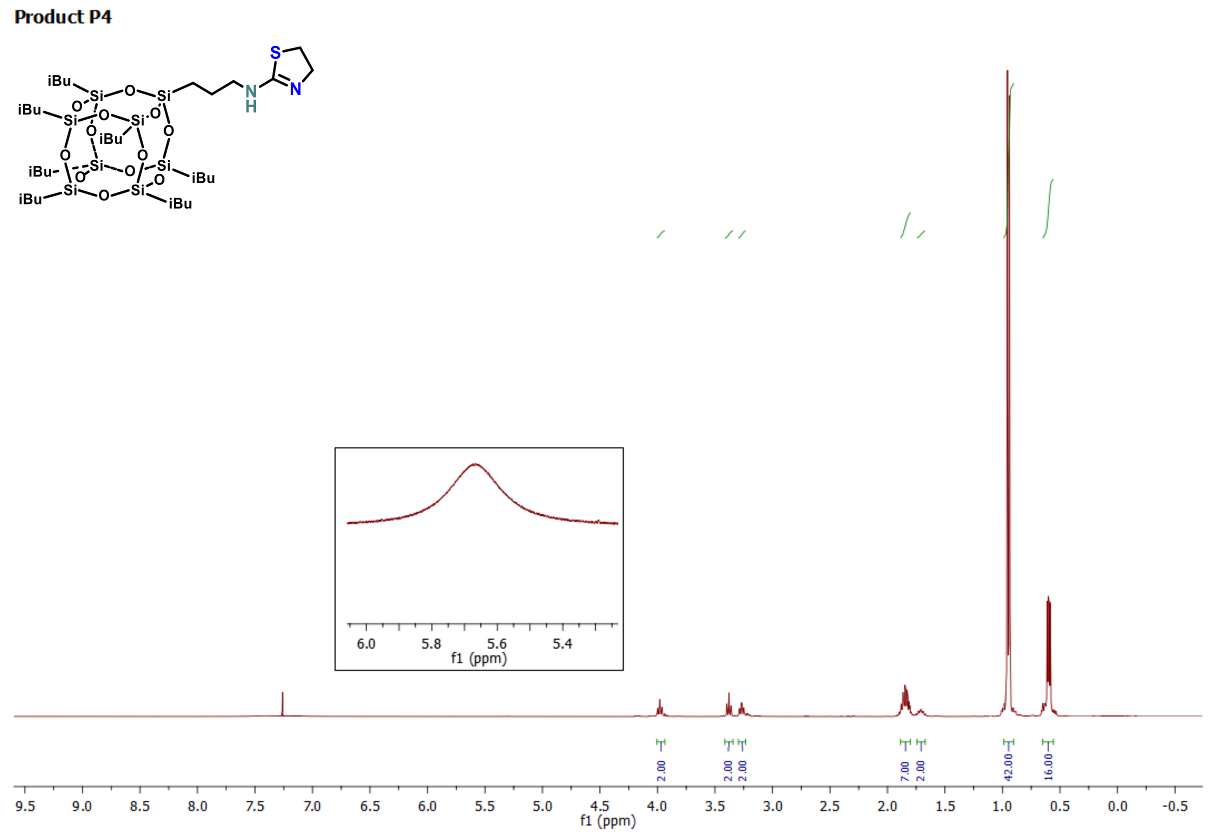

Figure S14. ^{1}H NMR (400 MHz, $CDCl_3$) of product **P4.** The N*H* signal visible after lifting the baseline.

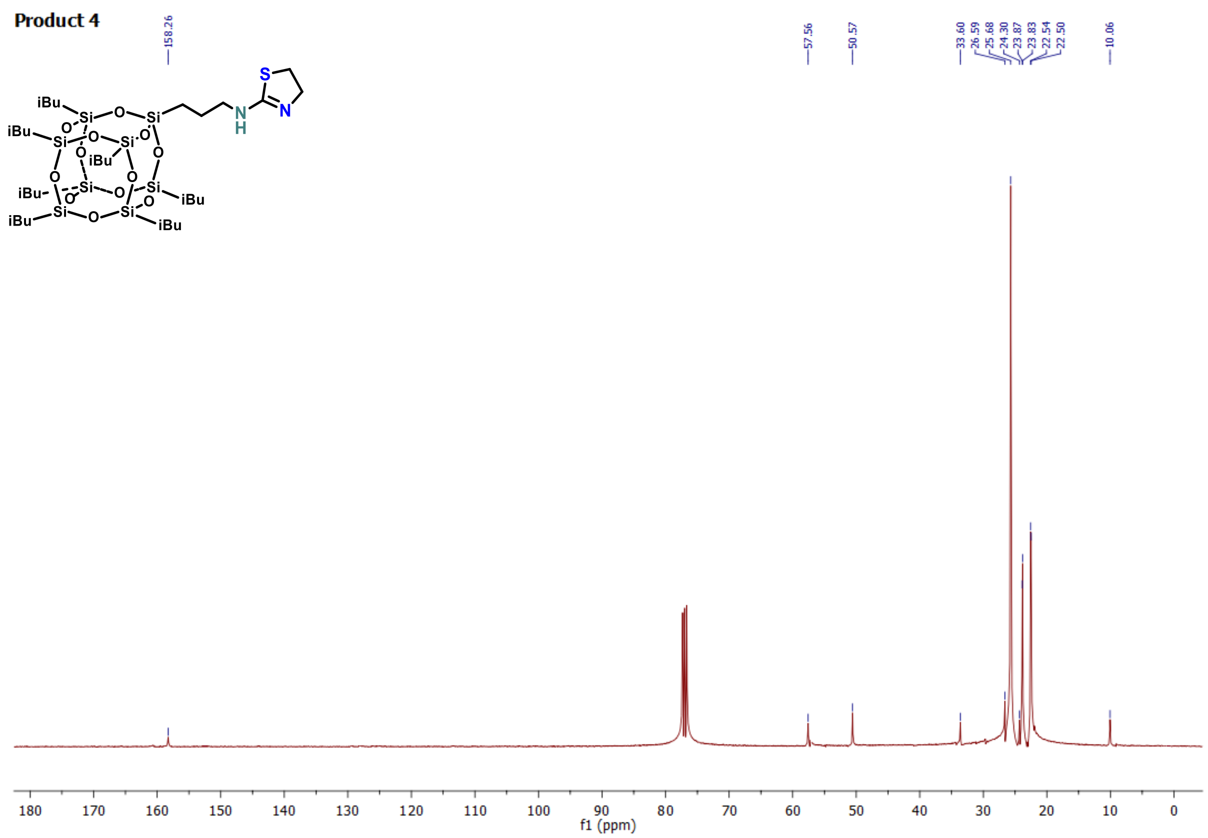

Figure S15. ^{13}C NMR (101 MHz, $CDCl_3$) of product **P4**

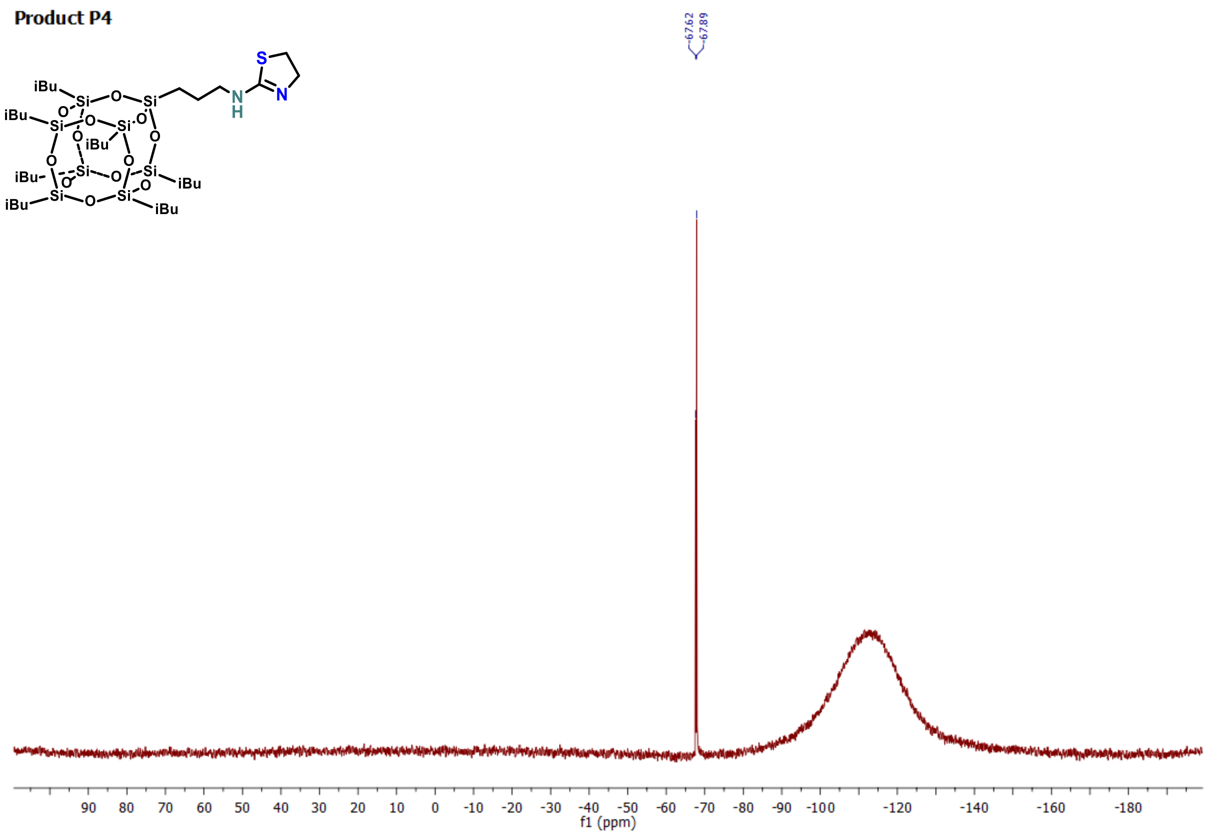

Figure S16. ^{29}Si NMR (79 MHz, $CDCl_3$) of product **P4**

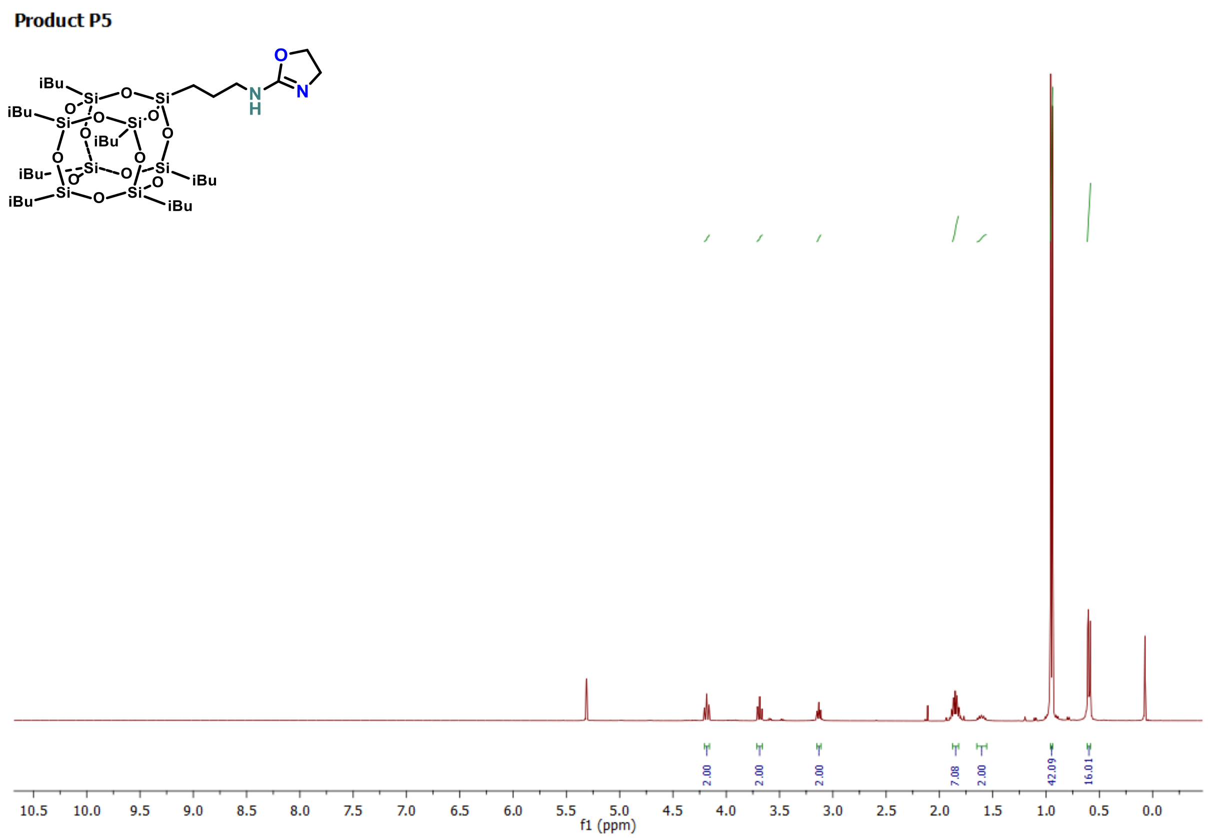

Figure S17. ^{1}H NMR (400 MHz, $CDCl_3$) of product **P5**

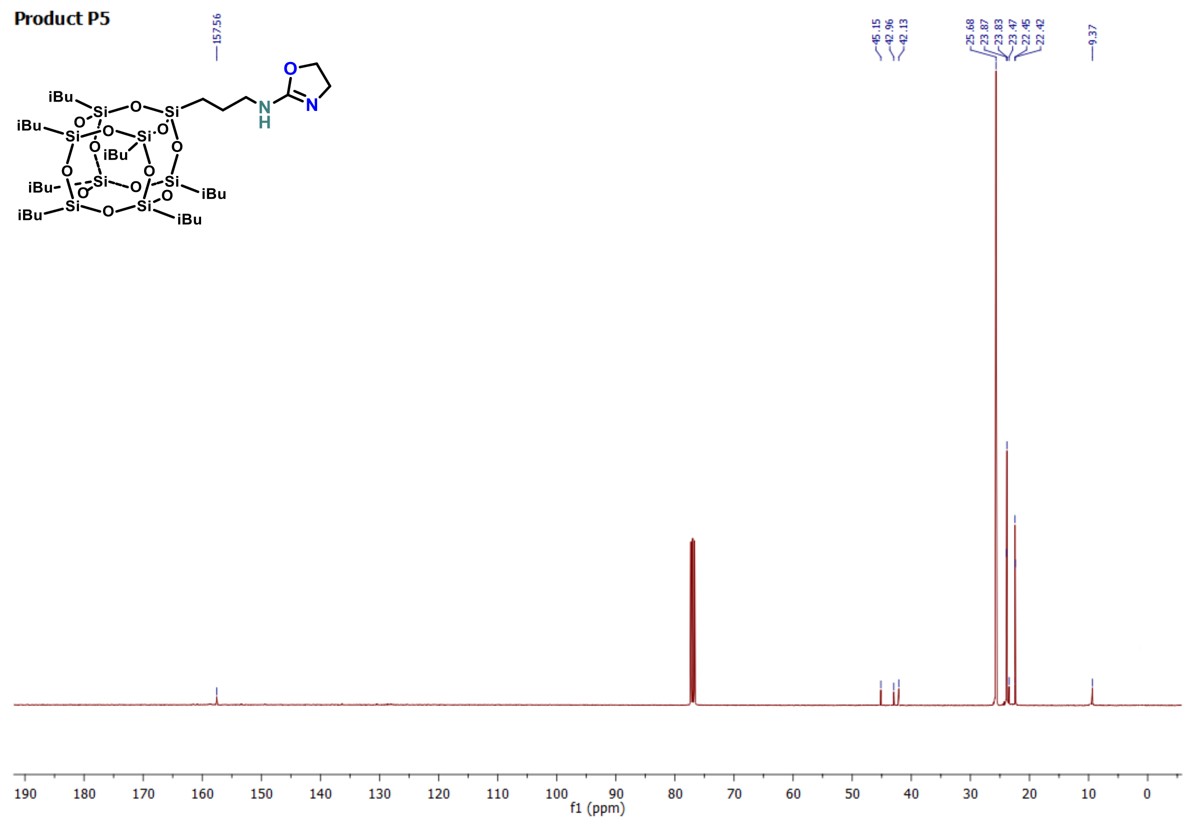

Figure S18. ^{13}C NMR (101 MHz, $CDCl_3$) of product **P5**

Product P5

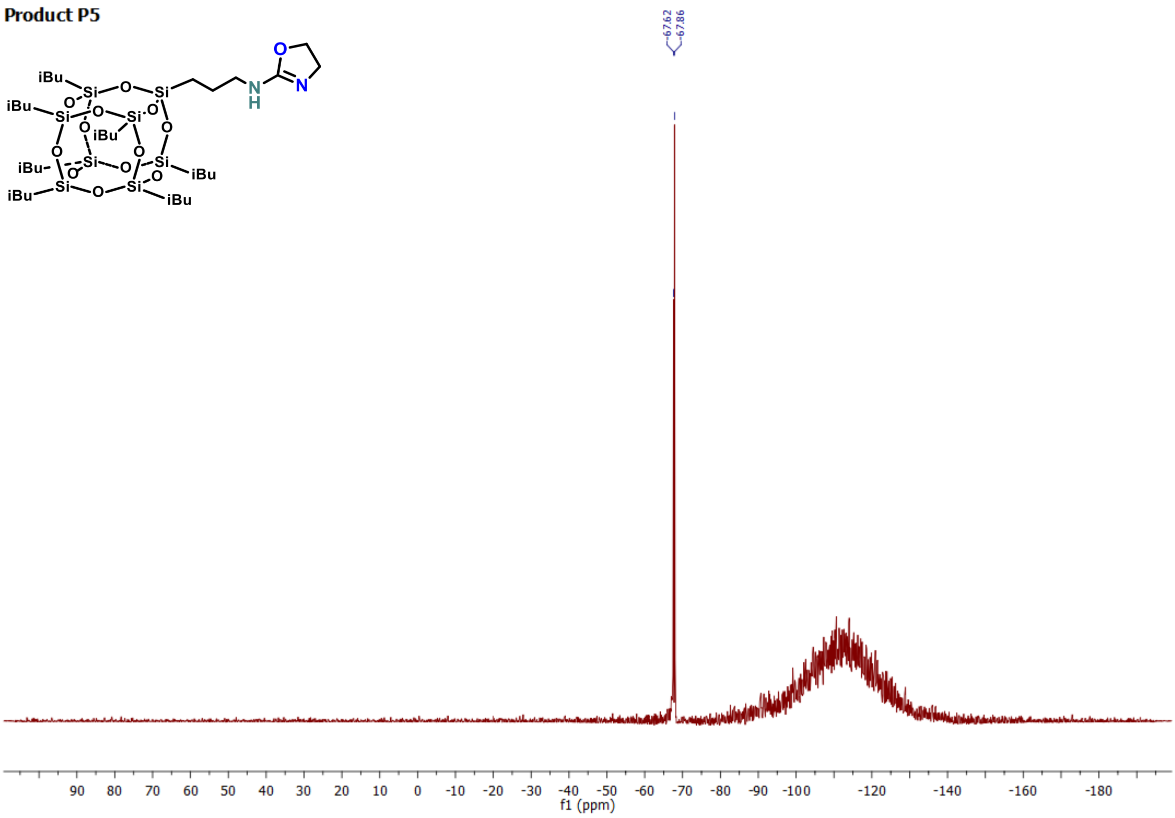

Figure S19. ^{29}Si NMR (79 MHz, $CDCl_3$) of product **P5**

Product P6

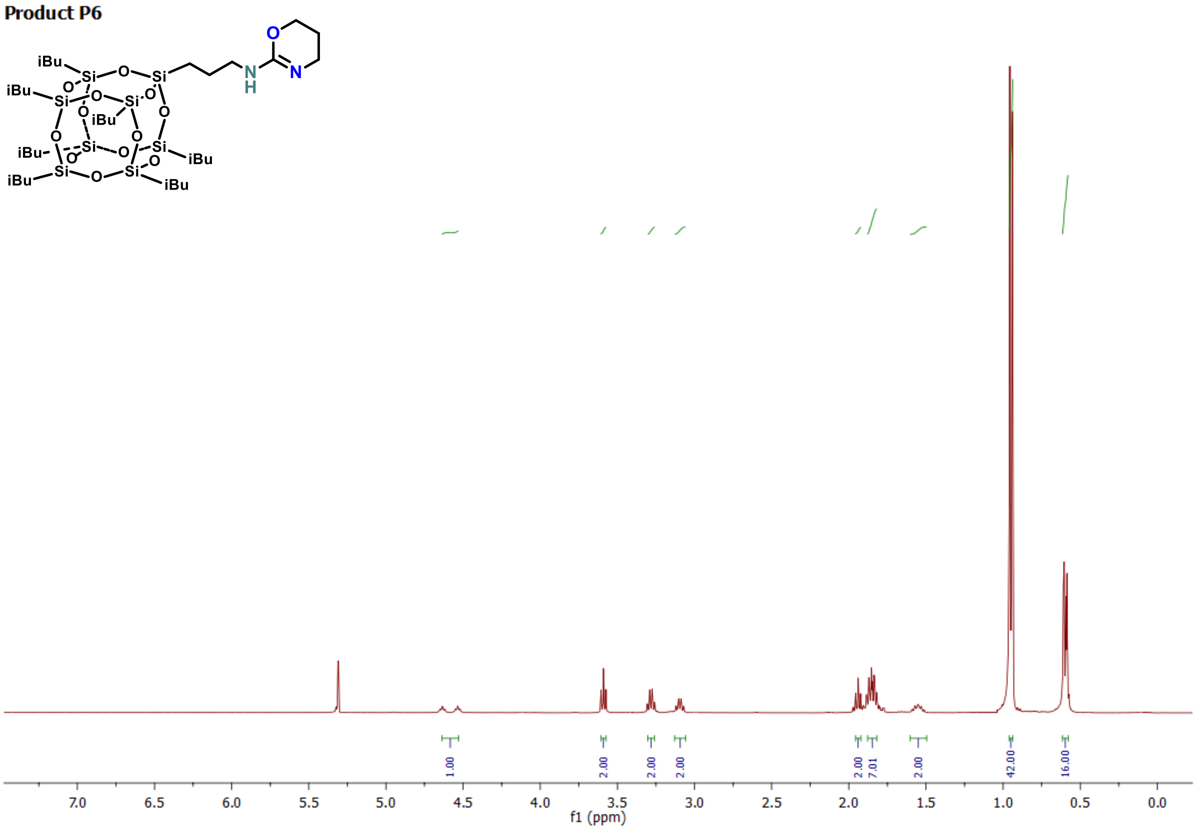

Figure S20. ^{1}H NMR (400 MHz, CD_2Cl_2) of product **P6**

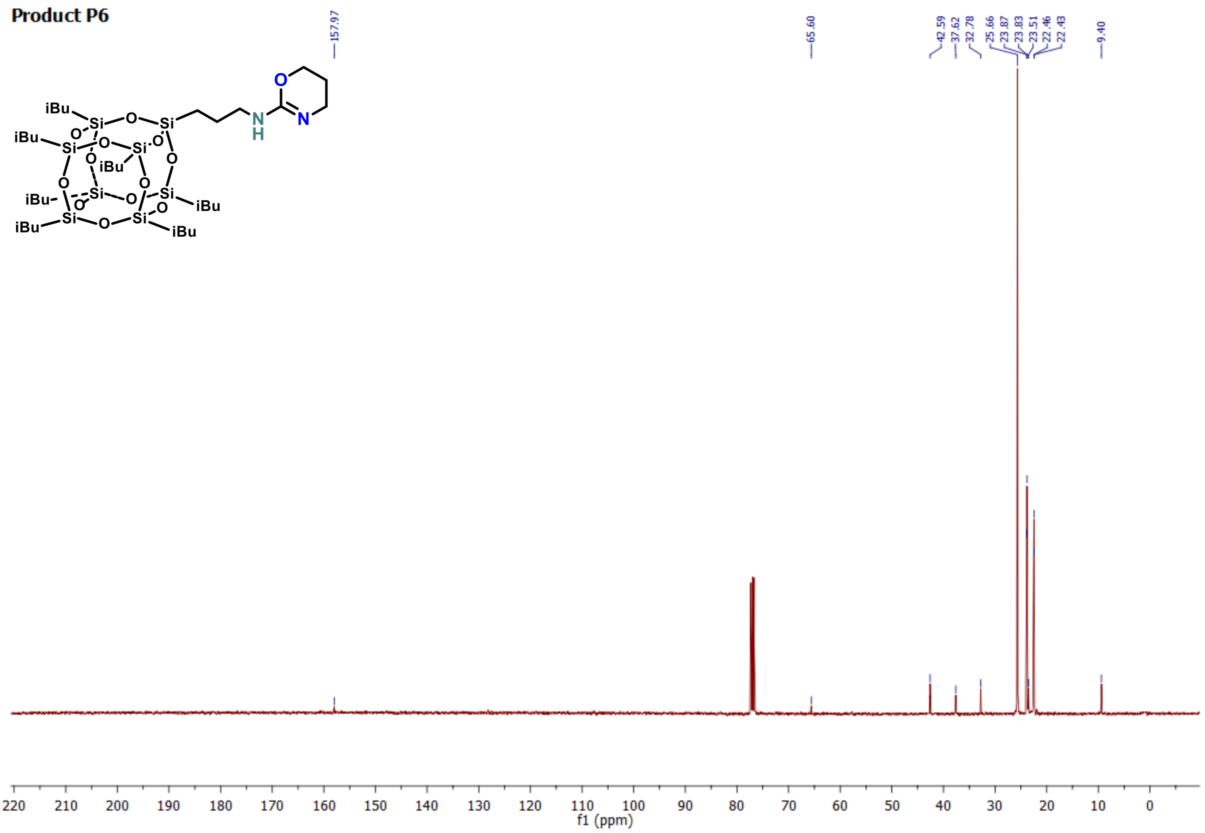

Figure S21. ^{13}C NMR (101 MHz, $CDCl_3$) of product **P6**

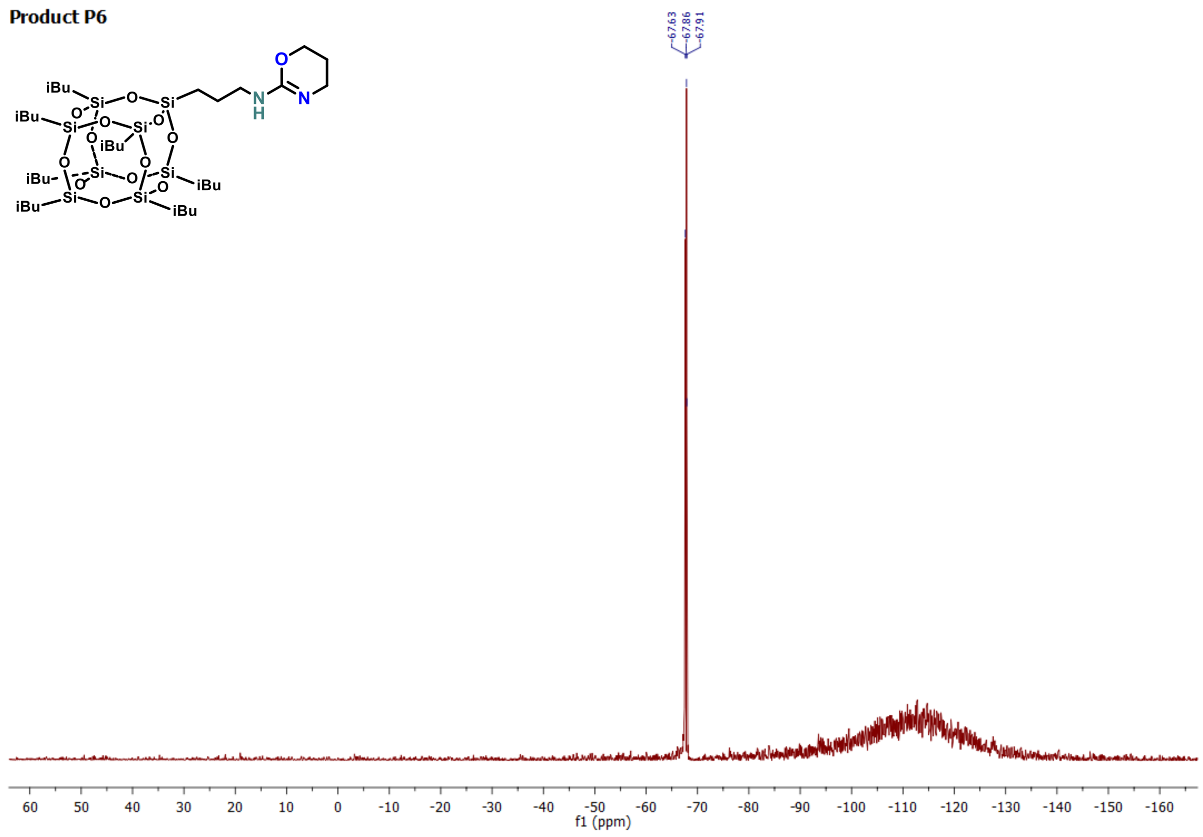

Figure S22. ^{29}Si NMR (79 MHz, $CDCl_3$) of product **P6**

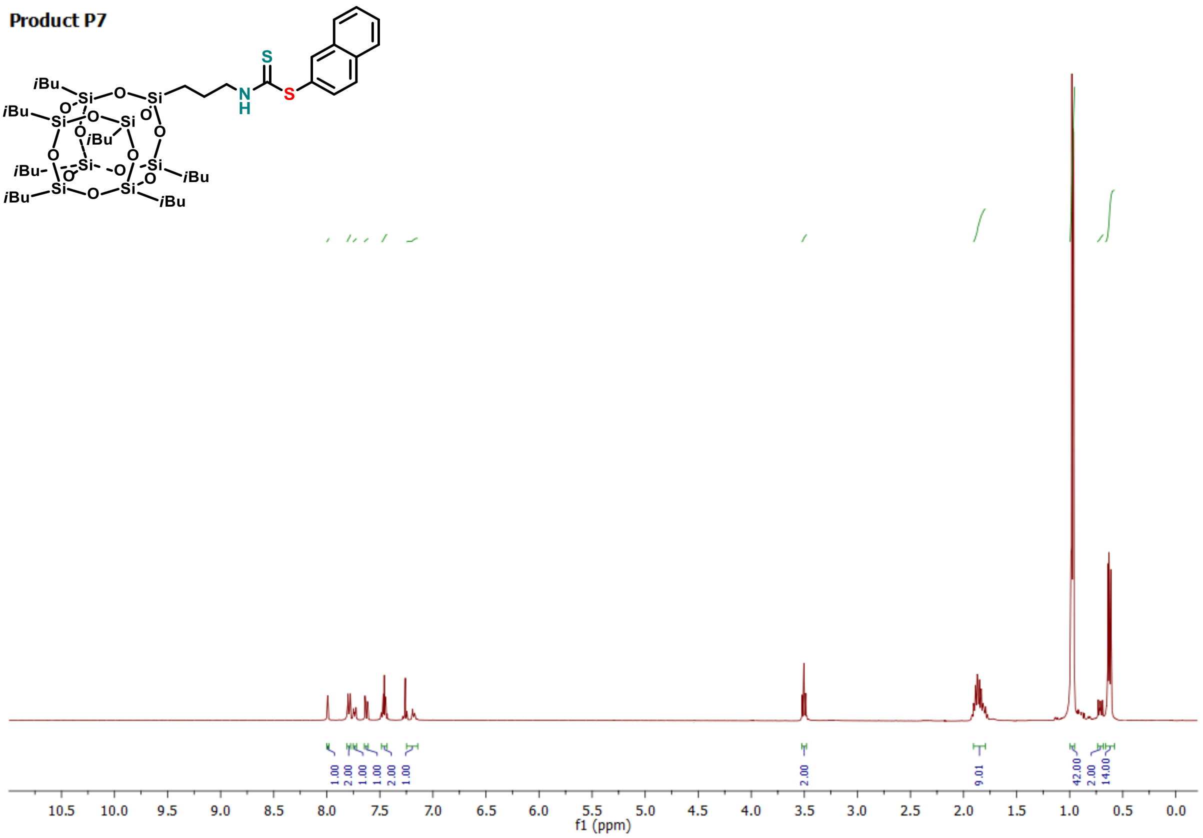

Figure S23. ^{1}H NMR (400 MHz, $CDCl_3$) of product **P7**

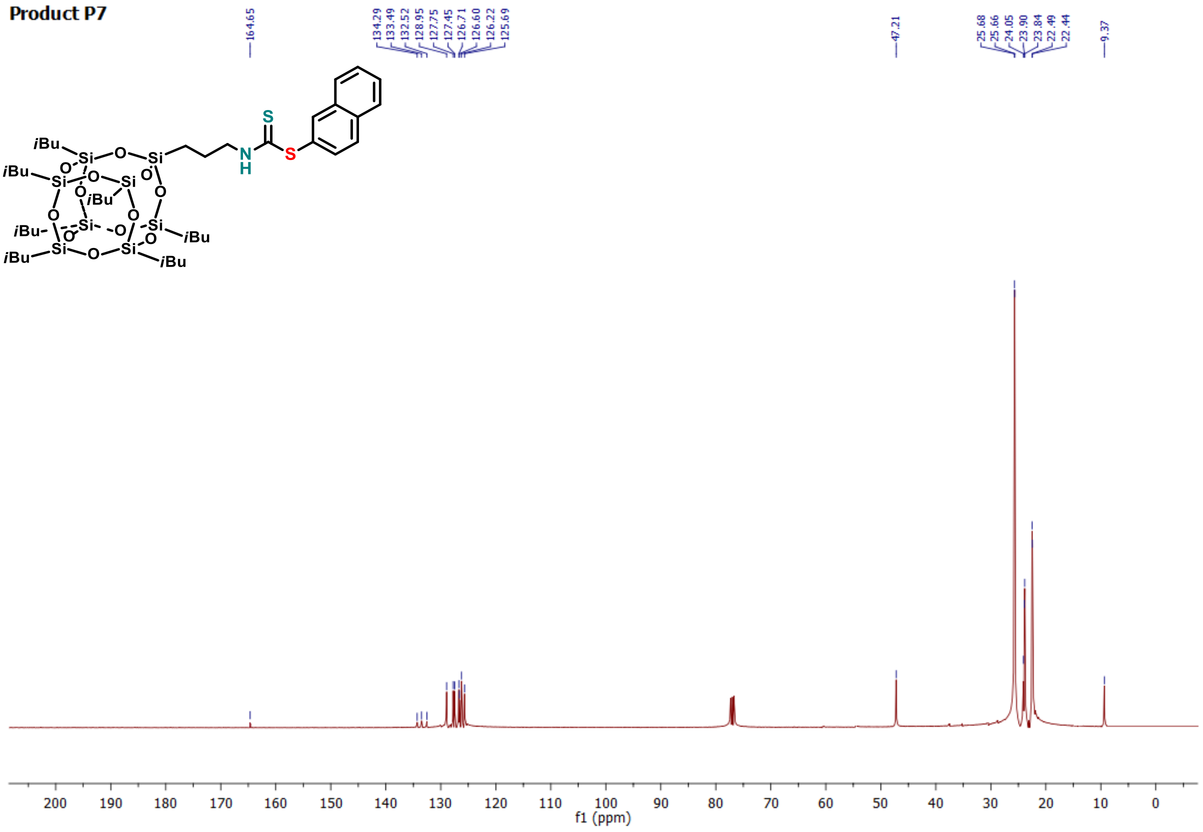

Figure S24. ^{13}C NMR (101 MHz, $CDCl_3$) of product **P7**

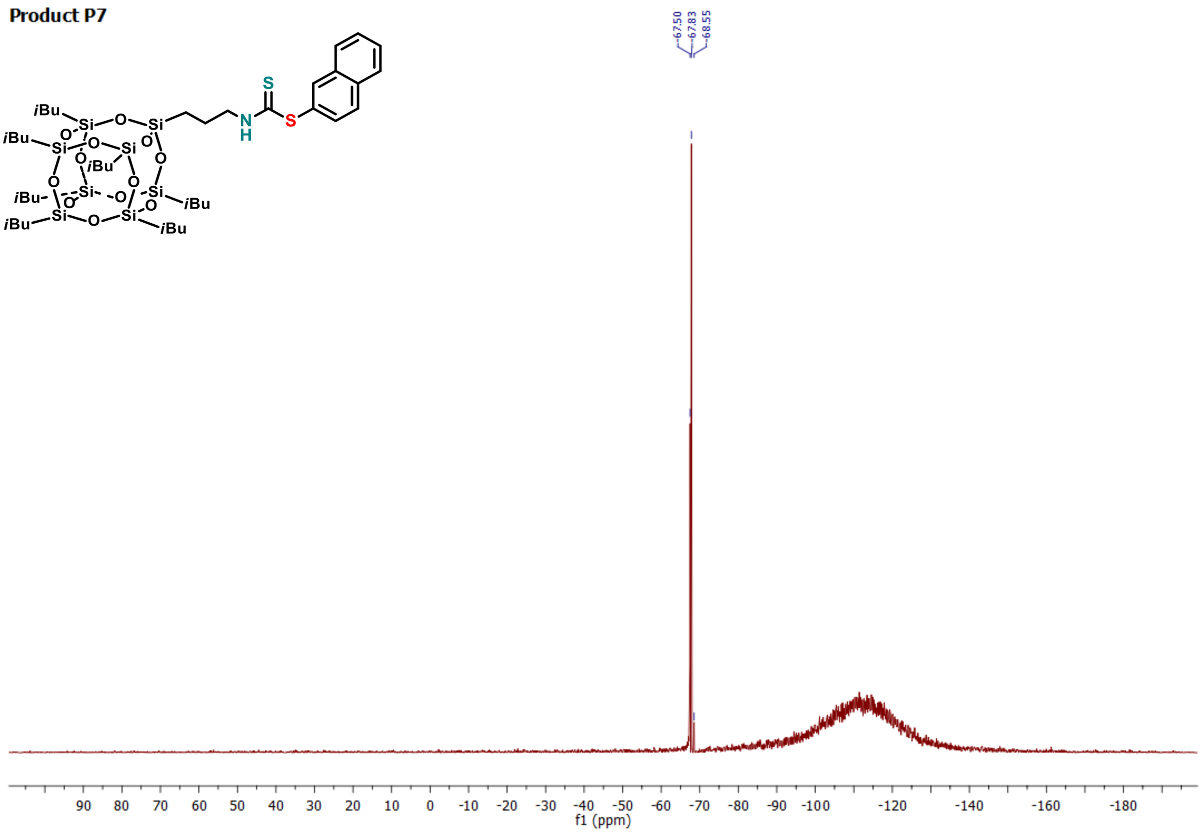

Figure S25. ^{29}Si NMR (79 MHz, $CDCl_3$) of product **P7**

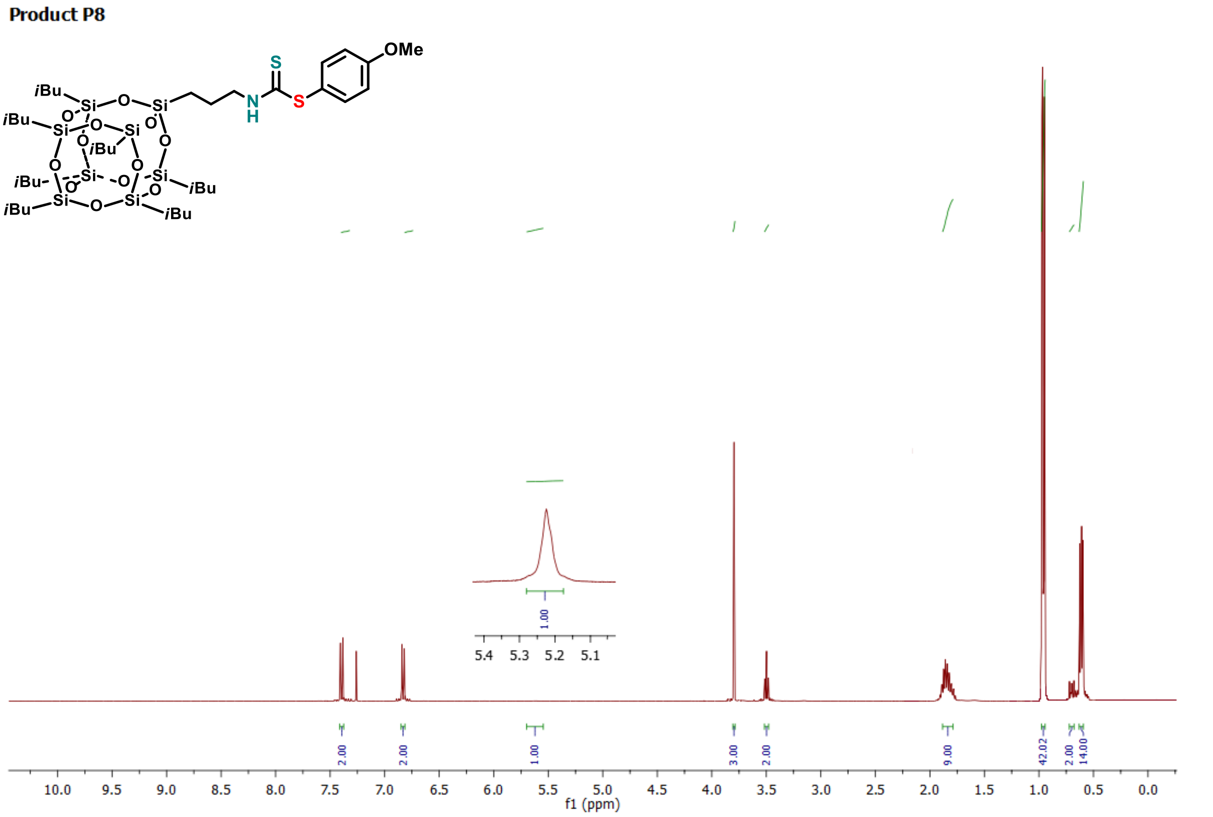

Figure S26. ^{1}H NMR (400 MHz, $CDCl_3$) of product **P8.** The N*H* signal visible after lifting the baseline.

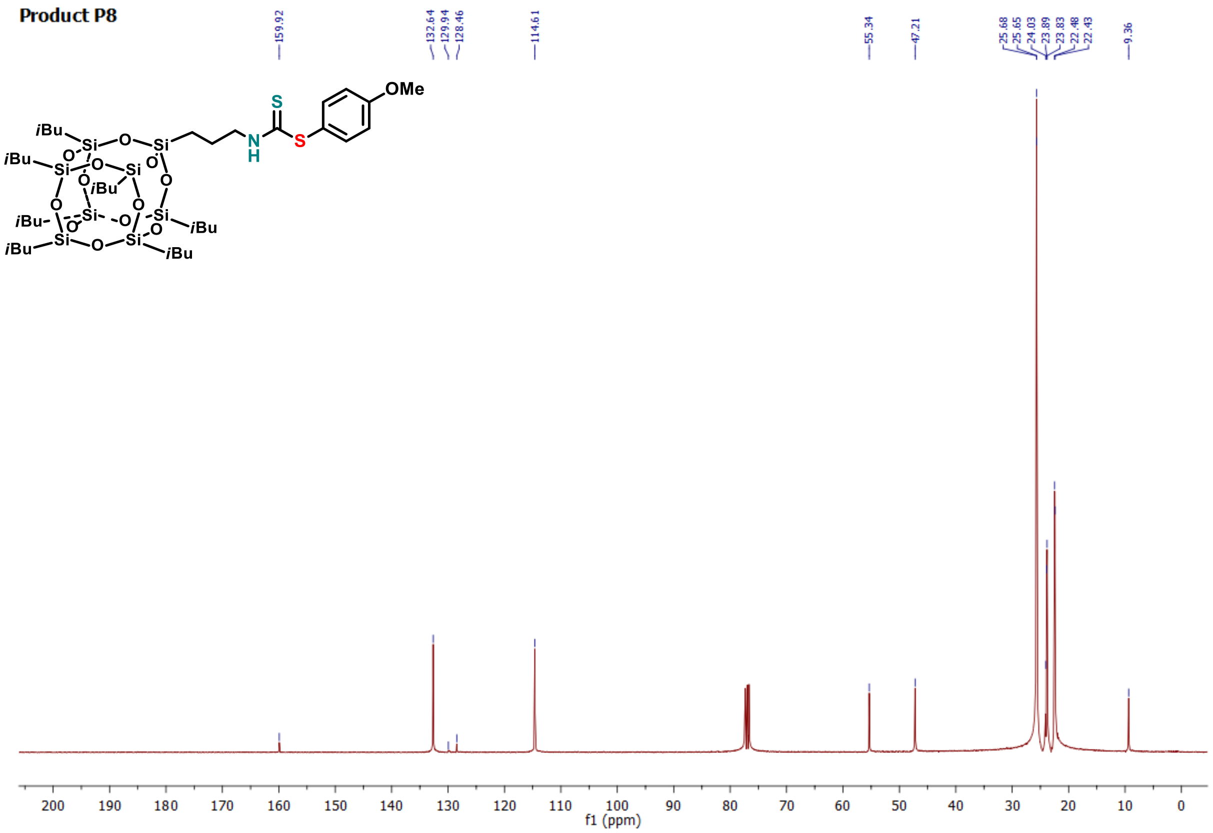

Figure S27. ^{13}C NMR (101 MHz, $CDCl_3$) of product **P8**

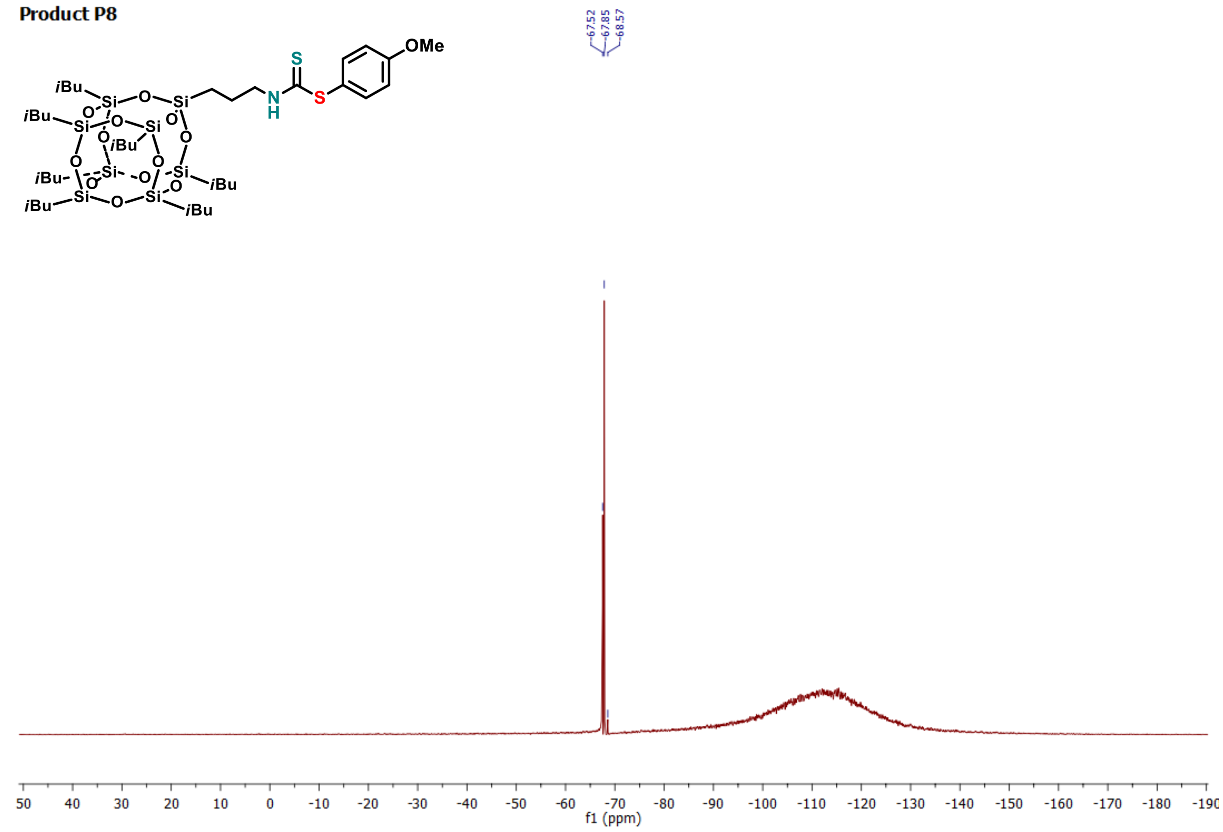

Figure S28. ^{29}Si NMR (79 MHz, $CDCl_3$) of product **P8**

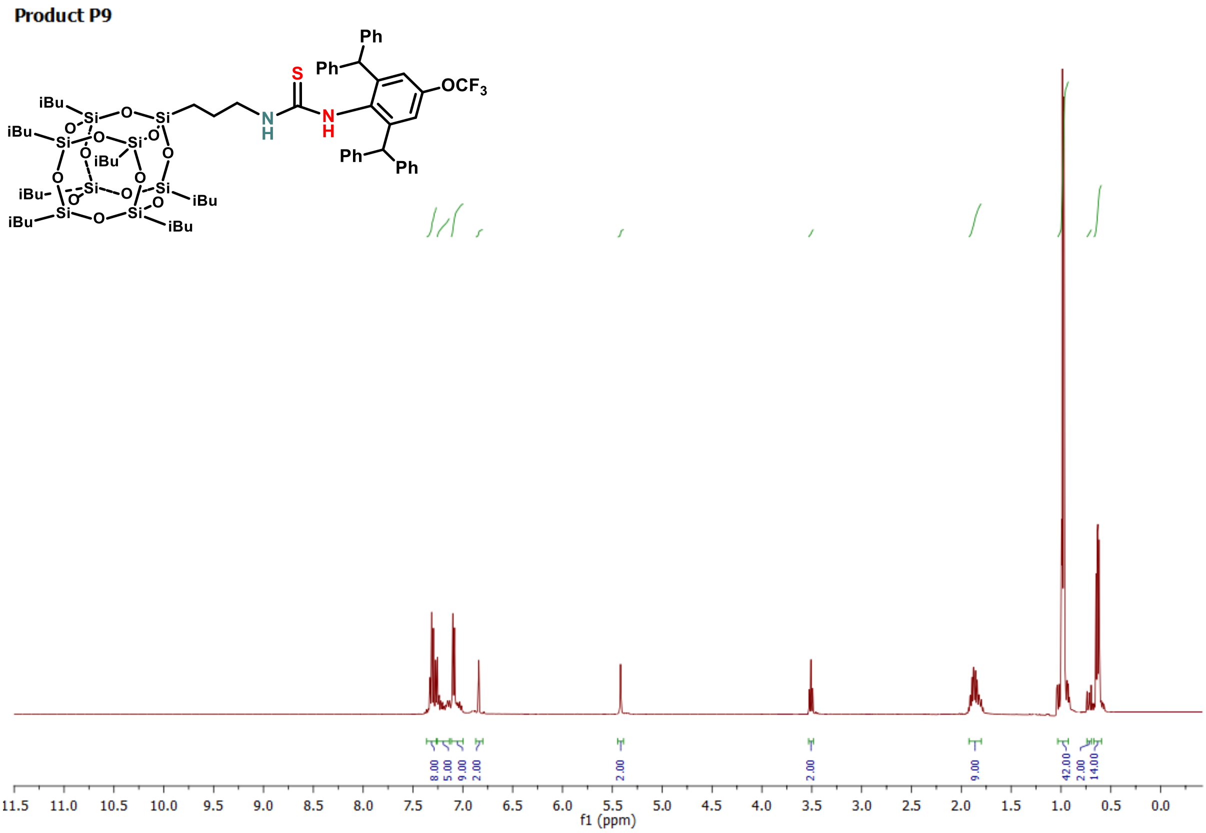

Figure S29. ^{1}H NMR (400 MHz, $CDCl_3$) of product **P9**

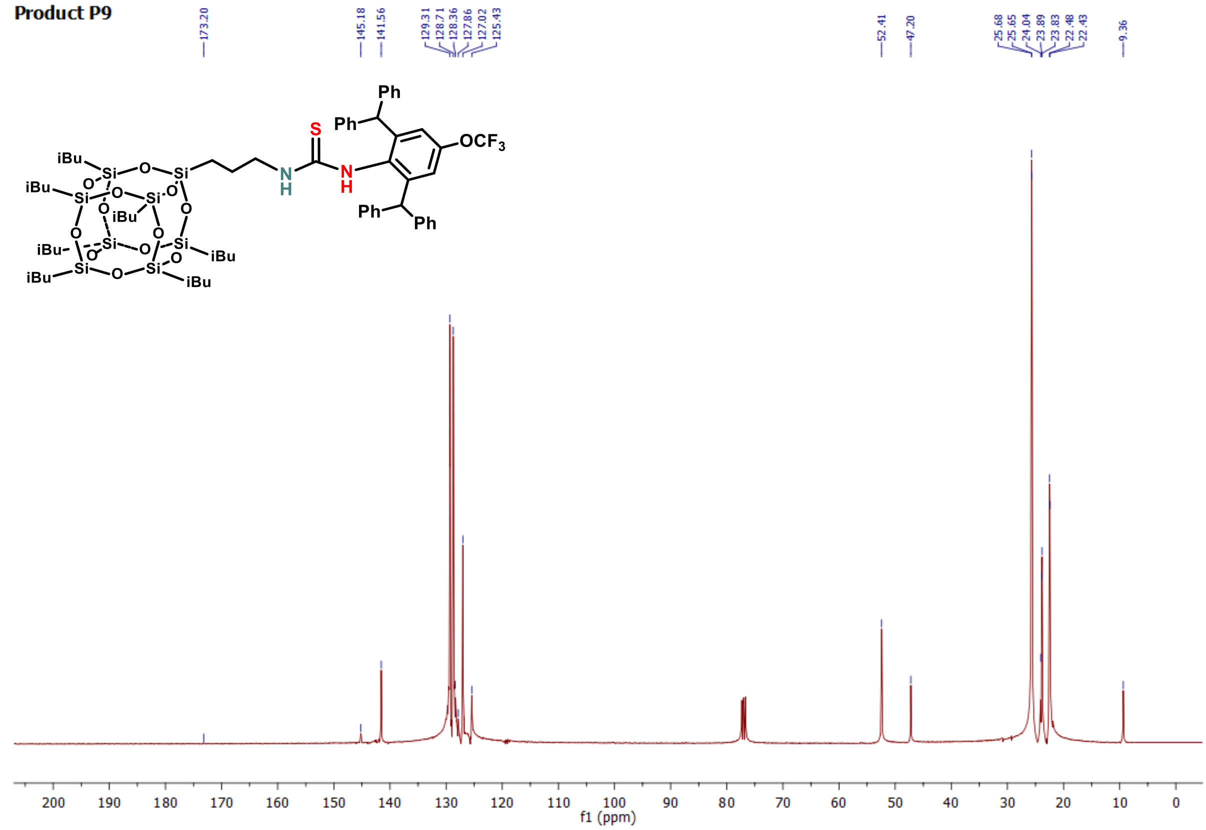

Figure S30. ^{13}C NMR (101 MHz, $CDCl_3$) of product **P9**

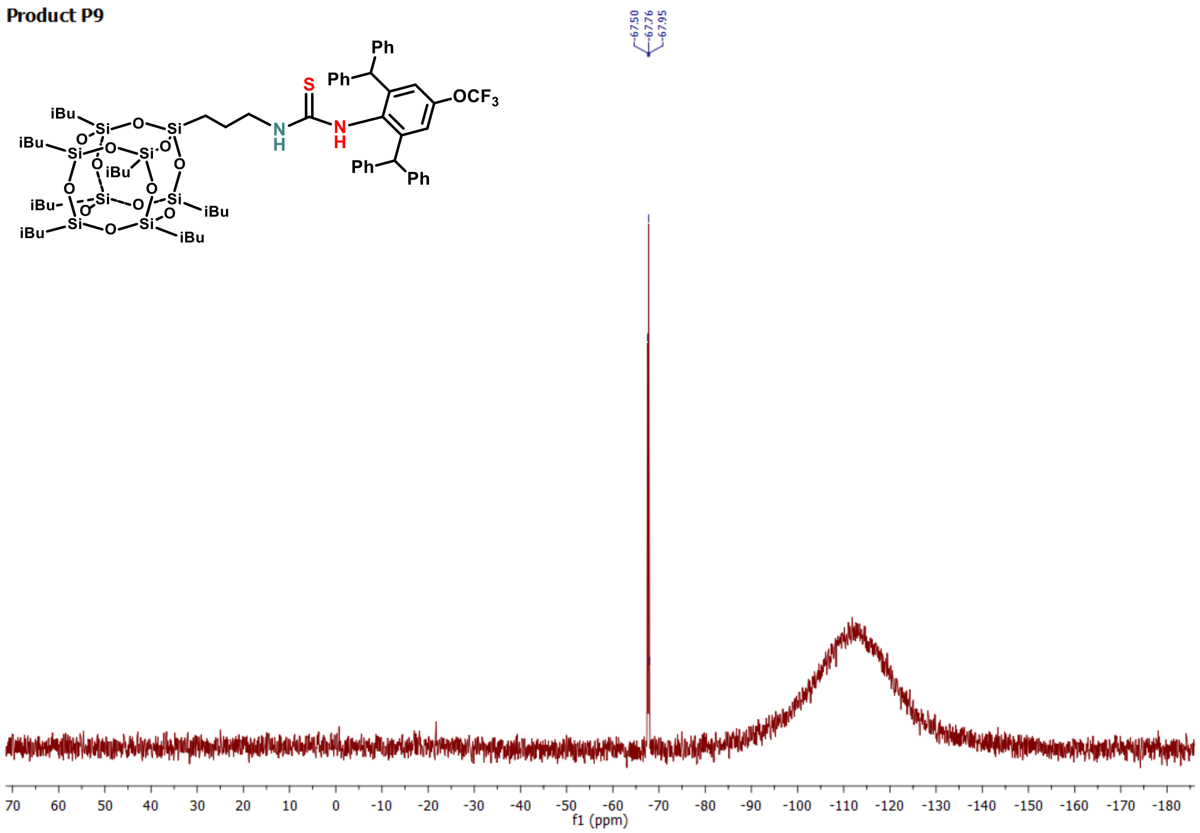

Figure S31. ^{29}Si NMR (79 MHz, $CDCl_3$) of product **P9**

5. Thermogravimetric analyses

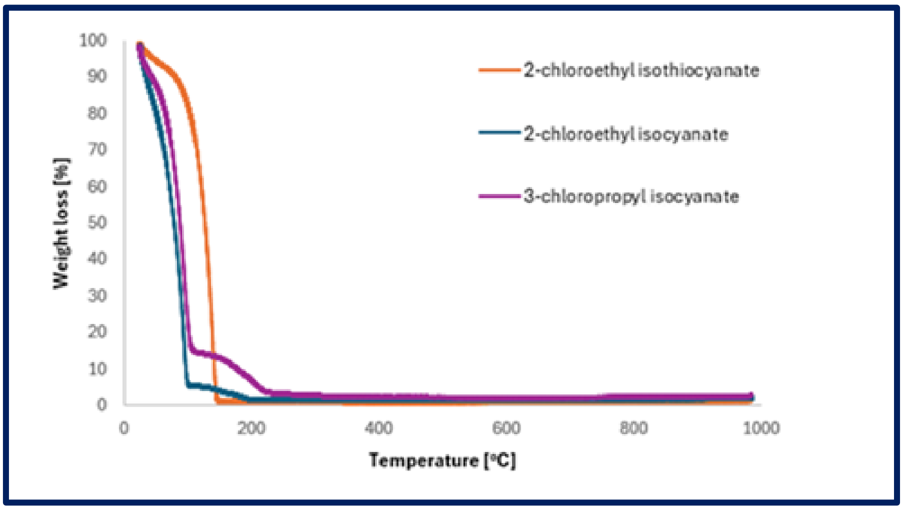

Figure S32. TGA curves of substrates

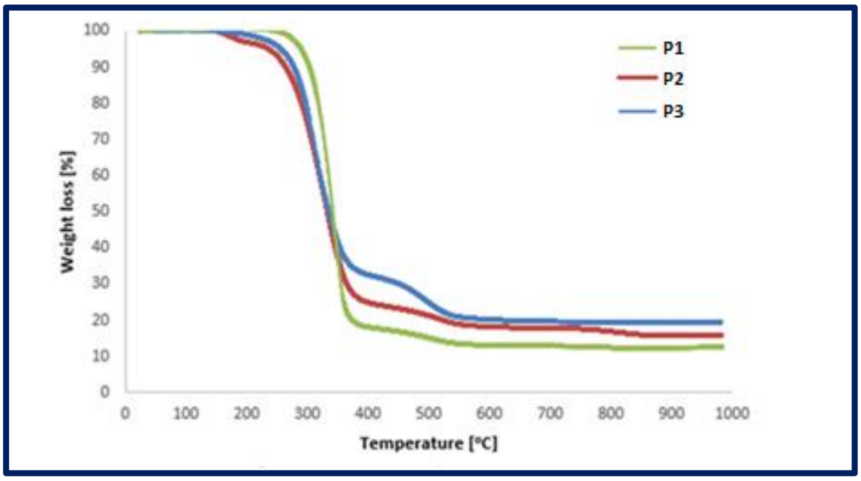

Figure S33. TGA curves of products **P1**-**P3**.

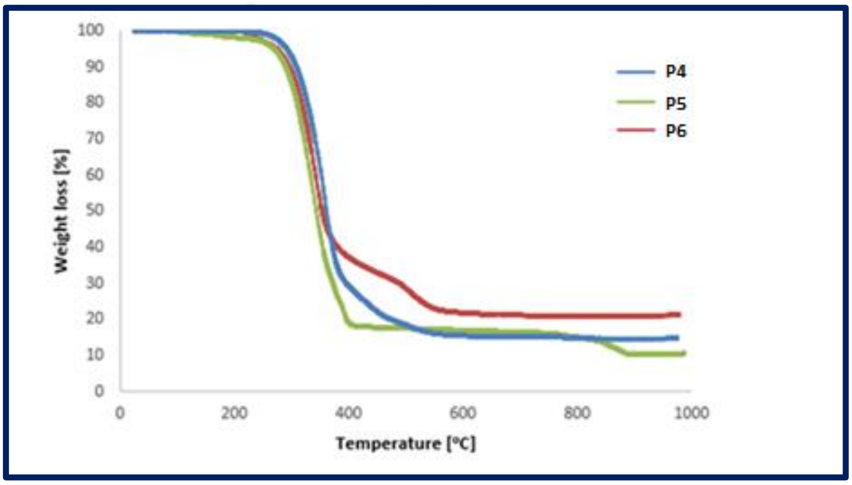

Figure S34. TGA curves of products **P4**-**P6**.

6. UV-VIS analyses of substrates

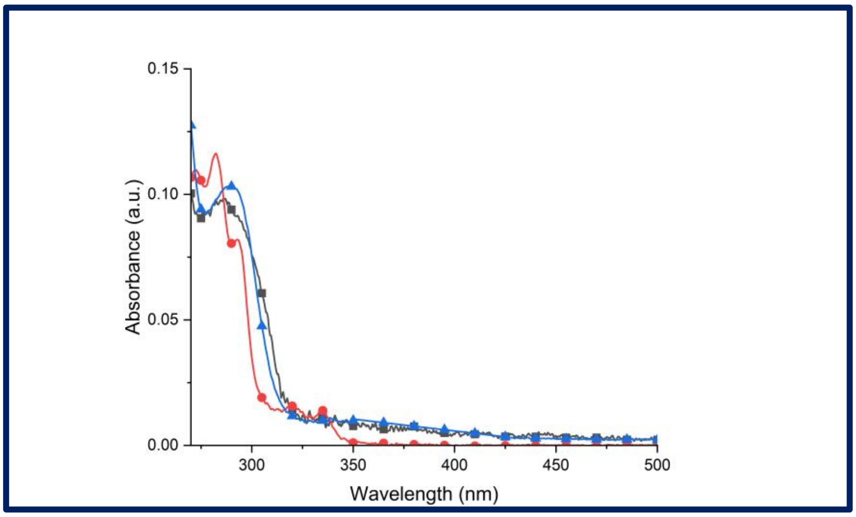

Figure S35. The UV-Vis absorption spectra of **2b** (■), **2a** (●) and **2c** (▲) measured in dichloromethane solutions at room temperature.

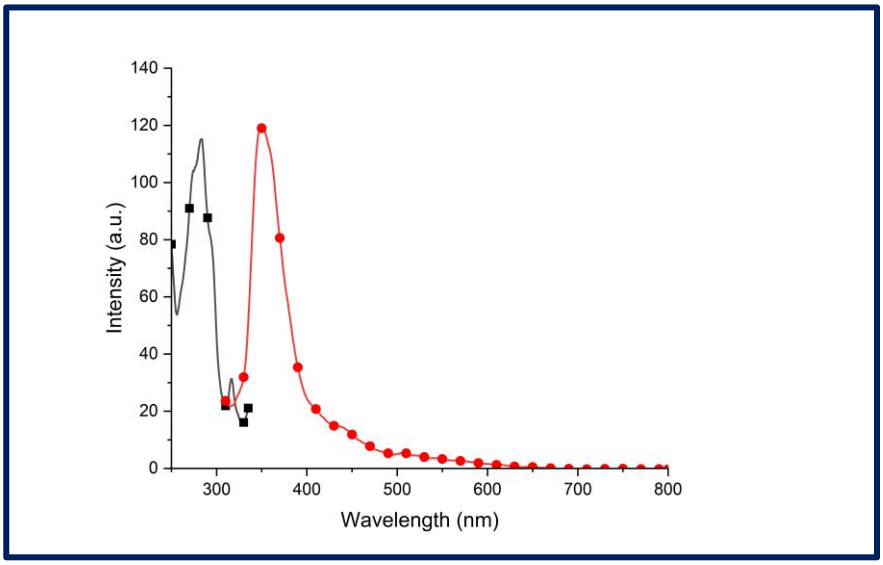

Figure S36. Excitation ($\lambda_{em.}$ = 348 nm ■) and emission ($\lambda_{ex.}$ = 282 nm ●) spectra of **2a** in dichloromethane.

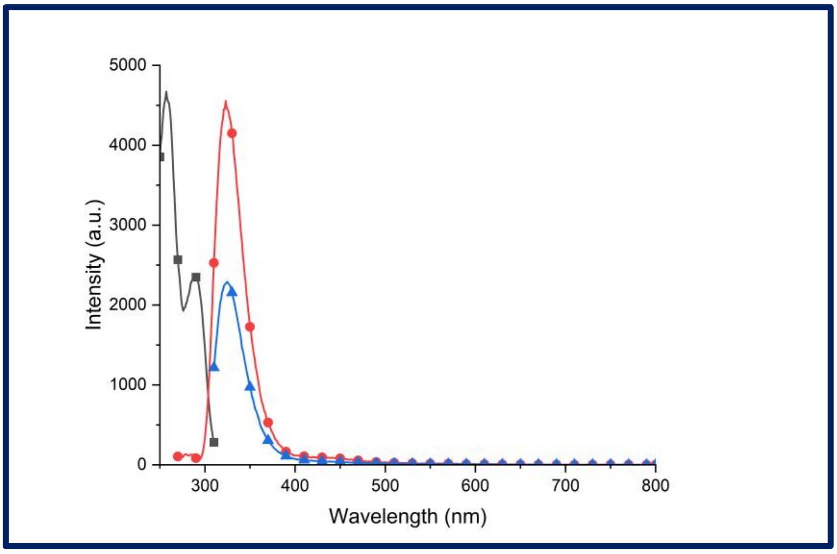

Figure S37. Excitation ($\lambda_{em.}$ = 325 nm; ■) and emission ($\lambda_{ex.}$ = 257 nm ● and $\lambda_{ex.}$ = 290 nm ▲) spectra of **2c** in dichloromethane.

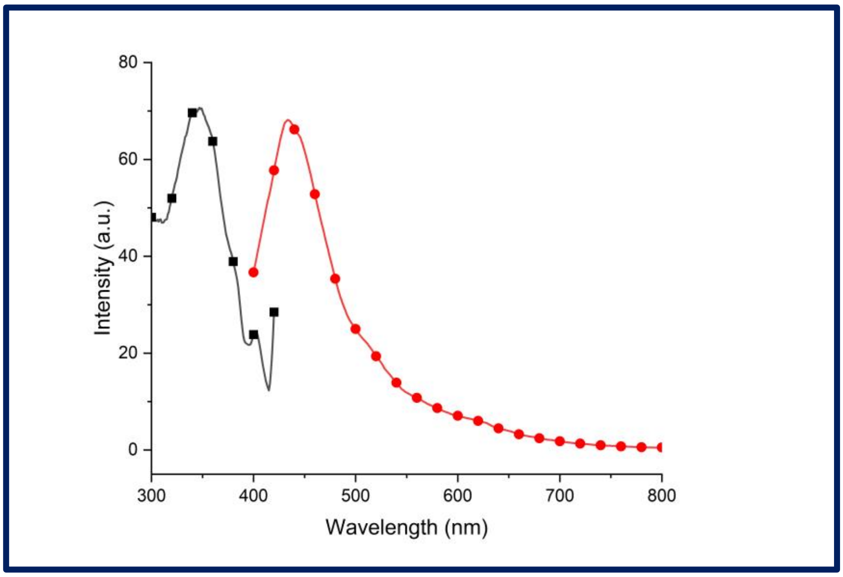

Figure S38. Excitation ($\lambda_{em.}$ = 435 nm; ■) and emission ($\lambda_{ex.}$ = 347 nm ●) spectra of **2c** in dichloromethane.

7. References

[S1] C. H. Wanke, J. L. Feijó, L. G. Barbosa, L. F. Campo, R. V. B. Oliveira, F. Horowitz, *Polymer*, **2011**, *52*, 1797-1802.
